# pH Jumps in a Protic Ionic Liquid Proceed by Vehicular Proton Transport

**DOI:** 10.1021/acs.jpclett.2c01457

**Published:** 2022-08-23

**Authors:** Sourav Maiti, Sunayana Mitra, Clinton A. Johnson, Kai C. Gronborg, Sean Garrett-Roe, Paul M. Donaldson

**Affiliations:** † Central Laser Facility, RCaH, STFC-Rutherford Appleton Laboratory, Harwell Science and Innovation Campus, Didcot OX11 0QX, United Kingdom; ‡ Department of Chemistry, University of Pittsburgh, 219 Parkman Avenue, Pittsburgh, Pennsylvania 15260, United States

## Abstract

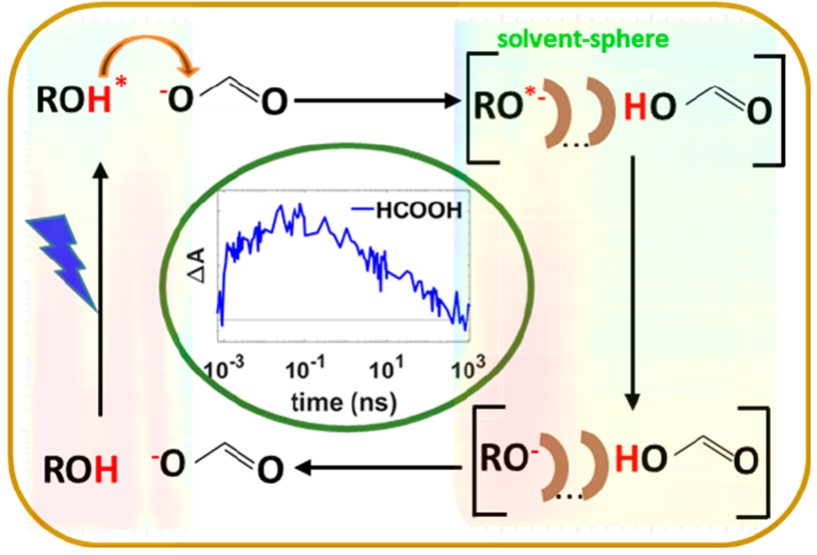

The dynamics of excess protons in the protic ionic liquid (PIL) ethylammonium formate (EAF) have been investigated from femtoseconds to microseconds using visible pump mid-infrared probe spectroscopy. The pH jump following the visible photoexcitation of a photoacid (8-hydroxypyrene-1,3,6-trisulfonic acid trisodium salt, HPTS) results in proton transfer to the formate of the EAF. The proton transfer predominantly (∼70%) occurs over picoseconds through a preformed hydrogen-bonded tight complex between HPTS and EAF. We investigate the longer-range and longer-time-scale proton-transport processes in the PIL by obtaining the ground-state conjugate base (RO^–^) dynamics from the congested transient-infrared spectra. The spectral kinetics indicate that the protons diffuse only a few solvent shells from the parent photoacid before recombining with RO^–^. A kinetic isotope effect of nearly unity (*k*_H_/*k*_D_ ≈ 1) suggests vehicular transfer and the transport of excess protons in this PIL. Our findings provide comprehensive insight into the complete photoprotolytic cycle of excess protons in a PIL.

Protic ionic liquids (PILs) are interesting nonaqueous solvents because of their nonvolatility, thermal and electrochemical stability, and high ionic conductivity. These may in principle raise the operating temperature of a fuel cell to >120 °C.^1,2^ PILs are salts that are molten at room temperature and formed by the reaction of a Brønsted–Lowry acid and a Brønsted–Lowry base. A mechanistic understanding of proton transport in PILs could help us to better realize their potential as proton-conducting materials for practical applications such as electrolytes for hydrogen fuel cells.^3^ Nevertheless, a great deal is unknown about the mechanisms of proton transport in PILs. Photoacids (ROH), compounds whose optical excitation leads to a transient increase in acidity (pH jump), have been utilized extensively as a trigger to investigate the proton transfer processes through time-resolved spectroscopy.^4−24^ Upon optical excitation, the acid dissociation constant, *K*_a_, of 8-hydroxypyrene-1,3,6-trisulfonic acid trisodium salt (HPTS, Figure 1(a)) increases almost 6 to7 orders of magnitude (a p*K*_a_ change from ∼7.4 to ∼1.4 in water),^5,6^ enabling the ultrafast release of protons into solution. This approach has provided valuable mechanistic insights into the proton transfer process in aqueous solvents for acid–base reactions. In the framework of the Eigen–Weller model, the bimolecular proton transfer reaction consists of a proton transfer step in a reactive complex followed by the diffusive separation of products.^7,17,22,25,26^

**Figure 1:**
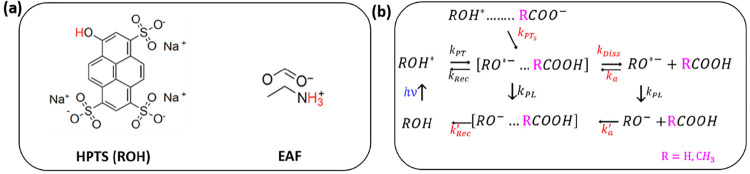
(a) Structure of 8-hydroxypyrene-1,3,6-trisulfonic acid trisodium salt (HPTS≡ROH) and ethylammonium formate (EAF). (b) Proposed photoprotolytic cycle of HPTS in water/acetate and EAF. Proton transfer from photoexcited HPTS (ROH*) to an acceptor (acetate or formate) can proceed reversibly via a direct hydrogen-bonded complex (“tight complex”) to form an excited encounter pair (EP*) with forward and reverse reaction rates *k*_PT_ and *k*_Rec_, respectively.^7,10−13,19,20,22^ A fraction of proton transfer occurs at a rate an order of magnitude slower (*k*_PTs_)) for species not initially hydrogen-bonded (“loose complex”).^7,10−13,19,20,22^ The EP* can dissociate (*k*_Diss_) into individual constituents (RO^*–^ and HCOOH) or generate (*k*_PL_), a ground-state EP through photoluminescence. The conjugate base RO^*–^ can reform the EP* bimolecularly with HCOOH (*k*_a_) or become de-excited (*k*_PL_) to RO^–^ through photoluminescence. The RO^–^ and HCOOH bimolecularly produce the ground-state EP (at rate *k*′_a_), whereupon proton transfer (*k*′_Rec_) from HCOOH to RO^–^ regenerates the HPTS, completing the cycle.

The transport of protons in ionic liquid media can be characterized by multiple approaches. Pulse-field gradient NMR techniques on imidazolium-based PILs can provide proton diffusion coefficients.^27−29^ Time-resolved photoluminescence, which has a typical time window of 0.1–15 ns, provides excited-state proton transfer dynamics from photoacids to the anion in PILs.^30,31^ Fuji et al. investigated the feasibility of proton transfer and the dynamics of associated intermediate states for naphthol-based photoacids to PILs with different anionic basicity.^31^ Recently, Thomaz et al. reported proton transfer dynamics from HPTS to aprotic solvent 1-methylimidazole (an important cation for ionic liquids) through time-resolved photoluminescence spectroscopy.^32^ Building upon the available literature,^7,8,10−13,19,20,22,30−32^ in this work, we are able to probe the full photoprotolytic cycle (Figure 1(b)) of HPTS by widening the accessible spectroscopic time scale. This enables us to investigate short-time-scale proton transfer (picoseconds) and long-time-scale proton transport (nanoseconds to microseconds).

We investigated proton transfer and transport in the PIL ethylammonium formate (EAF, Figure 1(a)) from femtoseconds to milliseconds (∼150 fs time resolution) using visible pump mid-infrared probe spectroscopy. The transient pump–probe measurements were performed in the time-resolved multiple probe spectroscopy (TRMPS) mode of operation, enabling measurement from femtoseconds to microseconds to milliseconds in a single experiment.^33^ Observing the complete photoprotolytic cycle has enabled us to determine both the ultrafast steps of proton transfer from the photoacid to the PIL and the long-range proton transport process (Figure 1(b)). Viewed as a hydroxyl compound, ROH, the photoexcitation of HPTS leads to a highly acidic excited state ROH*, resulting in a prompt, reversible proton transfer to formate in EAF and generating an encounter pair (EP*).^12,22^ The EP* dissociates reversibly into individual components RO^*–^ and formic acid (HCOOH) on the picosecond to nanosecond time scale. The EP* and RO^*–^ also relax to the electronic ground state through radiative and nonradiative pathways on a few nanoseconds time scale.^7,8,10−14,17,20,22^ The resulting ground-state species, RO^–^, then serves as a proton scavenger; it recombines with a proton to regenerate HPTS, completing the photoprotolytic cycle on the hundreds of nanoseconds time scale.

From our work, the key conclusions are as follows: (i) the proton transfer process predominantly proceeds through a “tight complex”, where HPTS is hydrogen bonded to formate prior to photoexcitation; (ii) the EP* is longer-lived in EAF compared to water owing to the higher viscosity of EAF; and (iii) proton transfer and transport follow a vehicular mechanism with no significant kinetic isotope effect.

HPTS (20 mM) dissolved in EAF was photoexcited (∼10 μm path length) with a 400 nm laser pulse (∼150 fs), and the resulting changes in the absorbance (Δ*A* = *A*_pumped_ – *A*_unpumped_, *A* = absorbance) were monitored in the mid-infrared region. The EAF synthesis and characterization are described in section 1 and Figure S1 of the Supporting Information (SI). The transient-infrared experiment has been described in detail elsewhere.^33^ A synchronized pump (400 nm, 1 μJ/pulse) and probe (1400–1900 cm^–1^, <0.1 μJ/pulse) operated at 1 and 10 kHz, respectively. The sample was rastered in the focal plane to avoid the degradation of HPTS. Figure 2(a) shows the transient-infrared spectra vs time 2D color map for the photoexcitation of HPTS in EAF at 400 nm.

**Figure 2:**
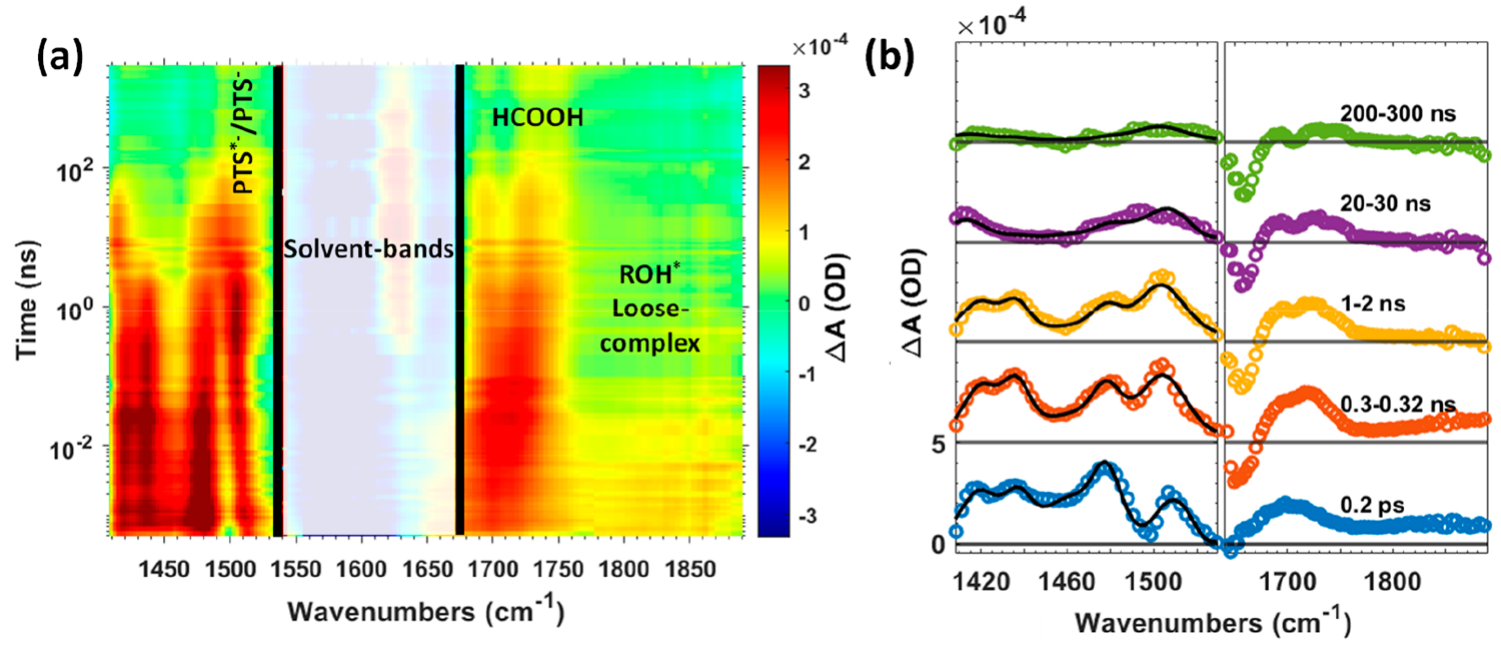
(a) 2D color map representing the transient absorbance (Δ*A*) of 20 mM HPTS dissolved in ethylammonium formate (EAF) upon 400 nm pump excitation (fwhm ∼150 fs). (b) Transient spectra at representative pump–probe delay times. The black lines are spectral line shape fits of the transient spectra, as discussed in the text. The apparent splitting of the formic acid band (∼1710 cm^–1^) accompanies the loss of excited-state species and results from thermal shifts of the adjacent, strong EAF absorption band (Figure S2, SI).

The transient-infrared spectra provide marker modes for the key species in the photoprotolytic cycle (Figure 2). The 1530–1650 cm^–1^ region is excluded from the analysis because strong infrared absorption from the formate carbonyl (−C=O) groups masks the transient-infrared signal. A photoinduced absorption band at ∼1725 cm^–1^ appears due to proton transfer from photoexcited HPTS (ROH*) to formate, creating formic acid.^7,10,12,13,22^ This feature follows the growth of the RO^*–^ photoinduced absorption at ∼1435 and ∼1503 cm^–1^. The formic acid absorption at ∼1725 cm^–1^ is free from overlap with other infrared bands at all pump–probe delay times, allowing the spectral amplitude of this species to be obtained simply by averaging the transient absorption band in the 1720–1735 cm^–1^ region. The broad photoinduced absorption blue of 1760 cm^–1^ is indicative of ROH*^–^formate loose-complex formation, which will be addressed in subsequent paragraphs.^12,13^

To isolate the kinetics of the involved species from the congested probe spectra, we fit the transient-infrared spectra in the 1425–1530 cm^–1^ region (primarily aromatic ring vibrational modes), with models for the infrared spectra of ROH, ROH*, RO^*–^, and RO^–^, each comprising a sum of Voigt line shapes^34^ (section 2, SI). The resulting spectra (black lines) are overlaid with the raw transient spectra signal amplitudes at selected frequencies, showing good agreement (Figure 2(b)). The models for the infrared spectra of these three species are determined by an analysis of the evolution of the transient spectra and by making use of spectra available in the literature (Figure S3, SI).^11−13,22^ Singular value decomposition (SVD) was applied to the kinetic scheme but failed to provide chemically meaningful components due to significant overlaps in frequency and decay time of the spectra of ROH*, RO^*–^, and RO^–^, especially RO^*–^ and RO^–^.

Figure 3(a,b) shows the kinetics of the formic acid (HCOOH) band and the extracted kinetics of RO^*–^ and RO^–^. The formic acid shows bimodal growth comprising a fast pulse-width-limited component and an order of magnitude slower component growing over ∼100 ps. Similar to HPTS/acetate studies in water, we attribute the fast-growth component to instantaneous (pulse-width-limited) proton transfer from photoexcited HPTS (ROH*) to formate in hydrogen-bonded tight complexes existing prior to photoexcitation (also supported by steady-state absorption spectra, Figure S1(c), SI).^7,22^ The relative amplitude of the fast-growth component accounts for the fraction of the tight complex (∼70%) from which the complexation constant^7,22^ of HPTS in EAF can be obtained as 0.21 M^–1^.

**Figure 3:**
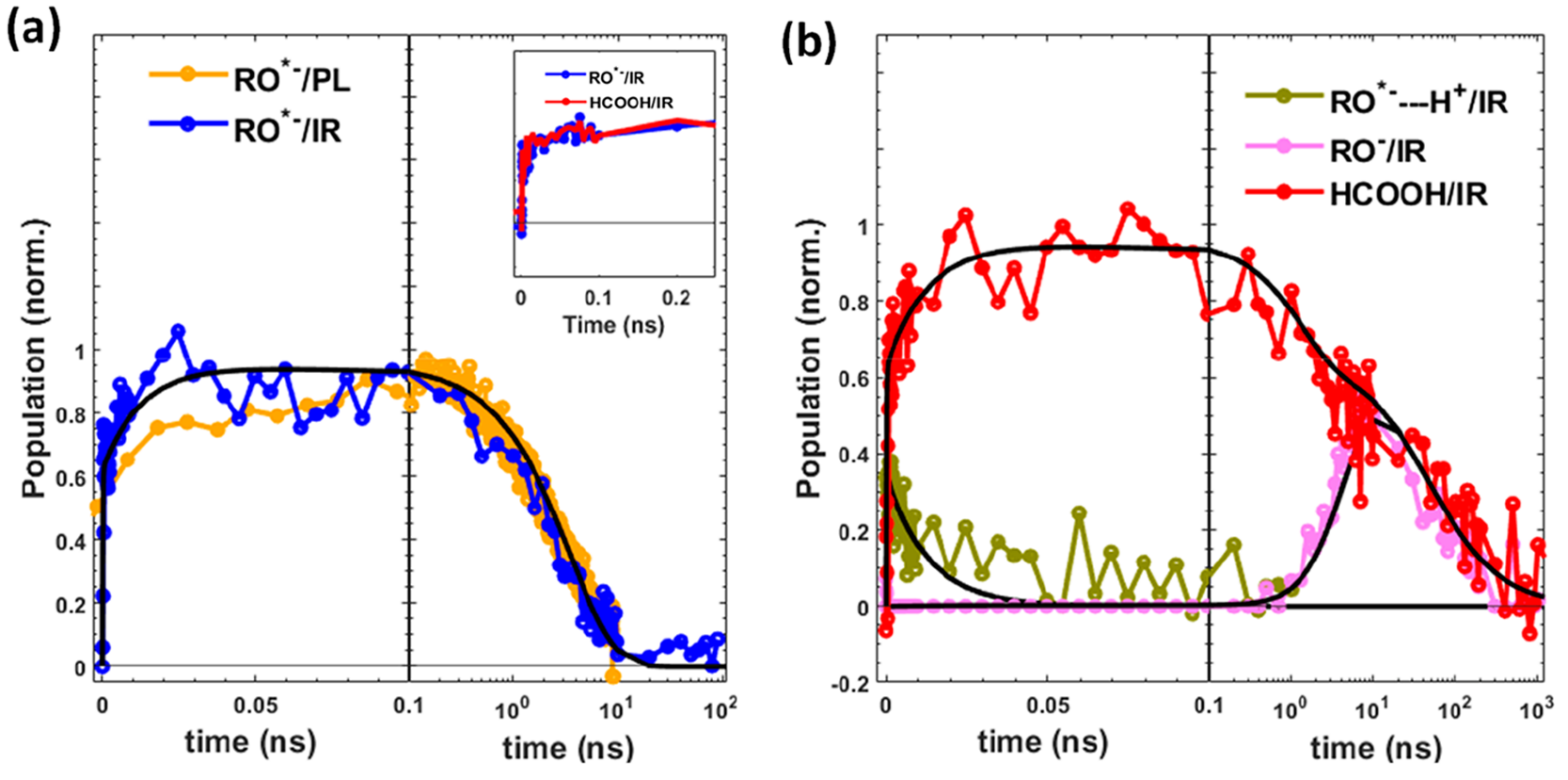
(a) Transient-infrared kinetics of RO^*–^ (blue) plotted along with the photoluminescence decay (orange). (b) Kinetics of loosely bound protons of ROH* (olive, average in the 1770–1850 cm^–1^ region), RO^–^ (pink), and formic acid (red, average in the 1720–1735 cm^–1^ region). For RO^*–^ and RO^–^, the kinetics are obtained from spectral model fitting of transient infrared absorption data (details in the text). The black lines represent fits according to the kinetic model of Figure 1(b). The inset in (a) shows early time comparisons of kinetics between HCOOH (1720–1735 cm^–1^) and RO^*–^ (1501–1505 cm^–1^).

The slower growth component can be ascribed to RO^*–^–formate pairs which are not strongly hydrogen-bonded (weakly complexed) at the time of photoexcitation and are termed as loose complex. In water, the loose complexes are solvent-separated encounter pairs.^7,12,13,22^ In the case of EAF, we attribute the loose complex to the RO^*–^–formate pairs with a different geometric configuration where fast proton transfer is unfavorable. These must reorganize to form a favorable configuration prior to proton transfer. Recent theoretical investigation of direct and solvent-mediated proton transfer from HPTS in water shows the importance of structural configuration on the proton transfer rates.^35,36^ Therefore, future theoretical studies in EAF can certainly help to decipher the exact configuration of the loose complex. The formic acid and RO^*–^ bands have identical growth dynamics (Figure 3(a), inset), suggesting that direct proton transfer occurs in both the tight and loose complexes. The growth of RO^–^ follows the decay of RO^*–^ (Figure 3). The RO^–^ decays by accepting a proton from formic acid to regenerate HPTS (ROH), and thus both RO^–^ and formic acid signals decay identically (Figure 3(b)).

We also observe a broad photoinduced absorption at frequencies blue of ∼1760 cm^–1^ in the transient-infrared spectra (Figure 2). In the case of HPTS in water, this has been attributed to loosely bound protons of ROH* as the O–H bond (Figure 1(a)) in ROH* weakens.^12,13^ The broad infrared absorption results from this loosely bound, highly polarizable proton. Based on this explanation, the broadband infrared absorption feature in HPTS/EAF likely results from similarly loosely bound protons of ROH* in the hydrogen-bonding environment of formate.

To corroborate our model, we compared the kinetics of RO^*–^ obtained from the spectral model fitting to the photoluminescence decay (Figure 3(a)) of RO^*–^ around its emission maximum of 510 nm (Figure S1, SI). The excellent agreement between the transient-infrared and photoluminescence measurements supports the accuracy of the infrared spectral line shape fits and interpretation.

The chemical kinetics scheme (Figure 1(b)) reproduces the observed population dynamics and provides quantitative estimates of proton transfer rates, ion-pair separation, and proton recombination (Table 1). A simultaneous fit of the loosely bound proton, RO^*–^, RO^–^, and formic acid kinetics through the nonlinear least-square method was used to yield best-fit parameters (section 3, SI). The populations obtained from the kinetic model (Figure 3, black lines) agree well with experimental data from picoseconds to microseconds. The bimodal formic acid and RO^*–^ growth is best estimated with a pulse-width-limited rise, *k*_PT_ ≈ 150 fs, followed by a slower growth component, *k*_PTs_, of 113.4 ns^–1^ (1/*k*_PT_s__ = 8.8 ps). The decay of the loosely complexed fraction of ROH*, *k*_PTs_, as indicated by the dynamics of the broadband infrared feature (1760–1850 cm^–1^), correlates with the slow growth component of formic acid (Figure 2(b)). Thus, after photoexcitation, the proton becomes loosely bound in ROH* but does not instantly transfer to formate, as in the direct complex. Structural rearrangements of the ROH* and EAF over longer time scales (tens of picoseconds) are required for the proton to transfer. About 90% of the photogenerated formic acid decays back to formate in ∼300 ns.

**Table 1:** Estimated Rate Constants Based on the Kinetic Model in Figure 1(b) for HPTS/EAF and DPTS/Deuterated EAF (EAF-3D)

rate constants^a^	HPTS/EAF	DPTS/EAF-3D	*k*_H_/*k*_D_
*k*_PT_^b^	6.67 × 10^3^ ns^–1^	6.67 × 10^3^ ns^–1^	
*k*_PTs_	113.4 *±* 1.2 ns^–1^	112.4 ± 0.98 ns^–1^	1.01
*k*_Rec_	396.5 ± 3.4 ns^–1^	401.4 ± 2.9 ns^–1^	0.99
*K*_Diss_	0.40 ± 0.01 ns^–1^	0.39 ± 0.01 ns^–1^	1.03
*k*_PL_^c^	0.30 ± 0.01 ns^–1^	0.29 ± 0.01 ns^–1^	1.03
*K*_a_^d^	0.5 × 10^10^ M ^–1^s^–1^	0.5 × 10^10^ M ^–1^s^–1^	
*K*′_a_	(3.27 ± 0.36) × 10^10^ M ^–1^s^–1^	(3.02 ± 0.24) × 10^10^ M ^–1^s^–1^	1.08
*K*′_Rec_	164.1 ± 1.5 ns^–1^	161.3 ± 1.3 ns^–1^	1.02

a The rates were optimized by taking reported values in the literature as a guide.^7,8,10−13,20,22,37−39^ The uncertainties represent the standard errors of the estimated parameters.

b Faster than the time resolution of the measurement.

c *k*_PL_ was optimized close to the photoluminescence lifetime.

d Because *k*_a_ is an order of magnitude smaller^19^ than *k*_Diss_, a constant rate was used for a better fit.

In order to determine the mechanism of proton transport, we determined the kinetic isotope effect (KIE = *k*_H_/*k*_D_) on the fitted rates through H–D isotope substitution. For HPTS in water, KIEs of  have been reported for the proton transfer rate (*k*_PTs_) to form the loose complex in the proton transfer cycle, suggesting the Grotthuss process, for which protons transfer from donor (ROH*) to acceptor (acetate) by hopping through water wires.^10,12,13,17,20,40,41^ The subsequent loss of the loose complex (*k*_Diss_) also shows a KIE of ∼1.5. We have examined DPTS/EAF (3D), where all of the exchangeable H were replaced with D, to record analogous transient absorption spectra (Figures S4 and S5, SI).

The kinetics of protonated and deuterated samples are nearly identical (Figure 4). The observed *k*_H_/*k*_D_ ≈ 1 was within the error for all rate constants (Table 1), suggesting vehicular proton transfer and transport at all stages of the photoprotolytic cycle. The growth of RO^*–^ is identical to the growth of formic acid (Figure 3(a)), suggesting direct proton transfer without hopping. Moreover, the decay of formic acid and RO^–^ is identical (Figure 3(b)), indicating a concerted process during the proton transport process. We have compared the photoluminescence decay of RO^*–^ in EAF and EAF-3D (Figure S5(c)), showing an identical decay with KIE ≈ 1, supporting the absence of proton hopping during the EP* decay process in EAF.

**Figure 4:**
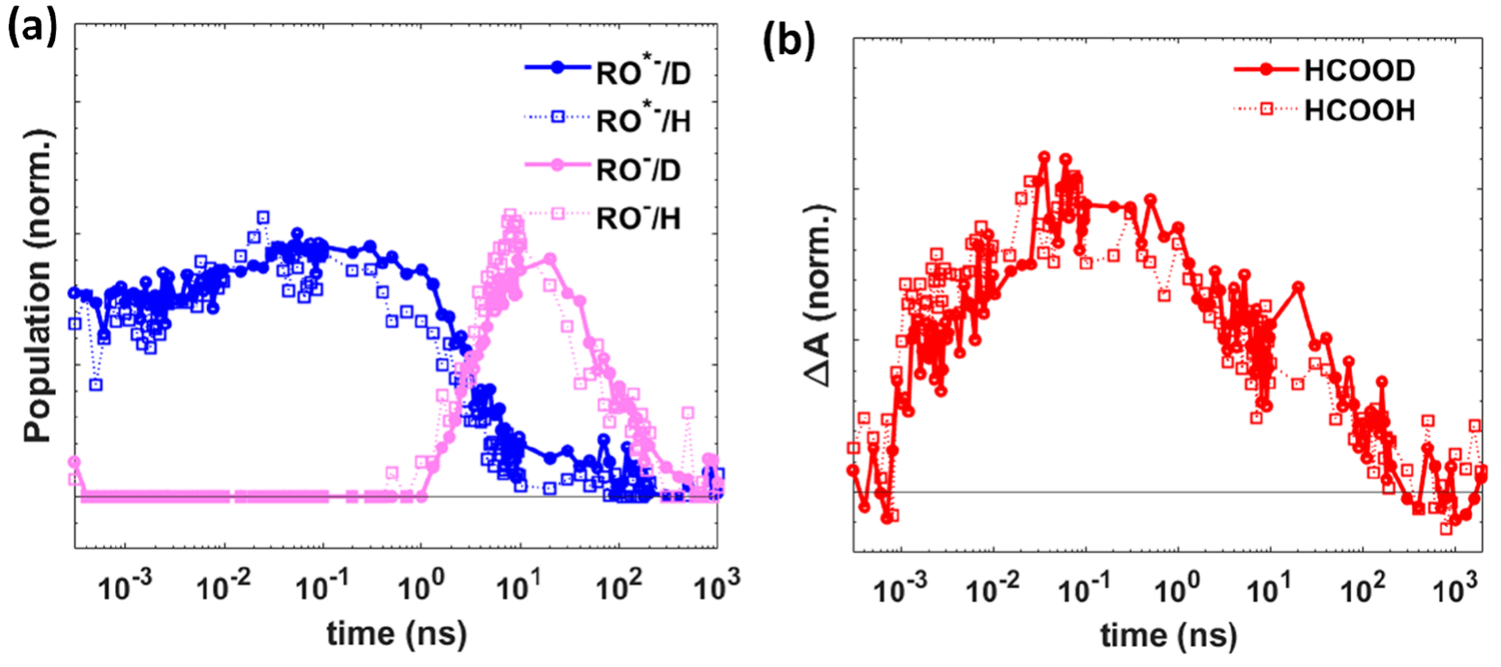
Comparison of the kinetics of (a) RO^*–^ and RO^–^ and (b) formic acid in HPTS/EAF (dotted line) and HPTS/EAF-3D (solid line). The populations of RO^*–^ and RO^–^ were obtained from spectral model fitting, whereas the population of formic acid was obtained from the average transient absorption between 1720 and 1735 cm^–1^.

How far does the photogenerated proton travel in EAF? We can estimate the excess proton diffusion length () using the combined diffusion coefficient (*D* = *D*_HCOOH_ + *D*_RO^–^_) for formic acid and RO^–^ in EAF and the lifetime (τ ≈ 300 ns) of the transient formic acid. The estimated diffusion length is ∼5 nm (section 4, SI). This distance corresponds to about ∼8–10 solvation shells, where one solvation shell in EAF is ∼0.5 nm, as determined by neutron diffraction.^42^

To understand the differences between EAF and aqueous systems, we compared the rates in the photoprotolytic cycle obtained for HPTS/EAF to equivalent measurements of HPTS/acetate (1 M) in D_2_O. The determination of p*K*_a_ in the ground and excited states of HPTS in EAF can give an idea about proton transfer rates compared to water.^21,24^ From steady-state absorption and photoluminescence data, we estimate a change of ∼6 p*K*_a_ units in EAF upon the photoexcitation of HPTS, which is similar to that in aqueous systems (section 5, SI). Following transient infrared measurements, the kinetics of the different species for the aqueous system were again obtained through spectral line shape fitting and kinetic modeling (Figure S6, SI) to obtain the rate constants (Table S1, SI).^7,8,10−13,20,22,37−39,43^ The proportion of the tight complex is higher in EAF (∼70%) than in water/acetate (∼40%) because the acceptor base (formate) is also the solvent in EAF and higher in concentration (11.4 M). Nevertheless, the RO^*–^ growth (and consequently the acetic/formic acid growth) rate constant is similar in water/acetate and EAF. The decay of EP* was ∼9 times slower in EAF than in water, which we attribute to the higher diffusion coefficient of acetic acid in water compared to that of formic acid in EAF. EAF’s higher viscosity (23.1 mPa s)^44^ compared to that of water (1 mPa s) likely leads to this slower EP* decay. RO^–^ is longer-lived in water, as the bimolecular association rate (*k*′_a_) of RO^–^ and acetic acid to form the EP in water (1 M acetate) is about 2 times slower than the association of RO^–^ with formic acid in EAF (Table 1 and Table S1, SI). The higher diffusion coefficient in water and Grotthuss transport can result in a larger separation between the acid and RO^–^, making the *k*′_a_ smaller in water.

Previous studies on proton diffusion in protic ionic liquids using pulsed-field gradient NMR have shown vehicular proton transport in equimolar (1:1) protic ionic liquids based on imidazolium cations and bis(trifluoromethylsulfonyl)imide anions.^27−29^ On the other hand, Grotthuss transport^40^ would be highly desirable in PIL-based fuel cells. Lin et al. have shown evidence that adding water (6 vol %) enhances the proton conductivity through Grotthuss transport in PILs with highly acidic cations (p*K*_a_ ≈ 0) such as 2-sulfethylmethylammonium triflate [2-Sema][TfO].^45^ On the other hand, Grotthuss transport as a proton transport mechanism has been demonstrated in pseudoionic liquids (equimolecular mixtures of *N*-methylimidazole and acetic acid).^46,47^ Recently, Ingenmey et al. have theoretically predicted combinations of substituted cations and anions for pseudoprotic ionic liquids that may display Grotthuss transport.^48^ Transient infrared spectroscopy will be an important tool for deciphering the proton transport mechanisms in these new PILs.

In conclusion, we have investigated proton transfer and long-range proton transport from the excited state of a photoacid HPTS in a protic ionic liquid ethylammonium formate through pump–probe vibrational spectroscopy. The proton transfer predominantly proceeds through hydrogen-bonded HPTS-EAF complexes having an ultrafast proton transfer rate (<150 fs) whereas proton transfer in a proportion of loosely complexed pairs is an order of magnitude slower. The long-range proton transport was deciphered by analyzing the kinetics of both excited- and ground-state species with transient-infrared and photoluminescence, suggesting proton transport to a few (∼10) solvent shells in the protic ionic liquid. The absence of the kinetic isotope effect suggests the presence of vehicular transfer and the transport of excess protons in EAF across the photoprotolytic cycle. The wide time-range study presented here improves on transient-infrared spectroscopic approaches for studying the solvent influence on whole photoprotolytic cycles, paving the way to investigate long-range excess proton transport in additional PIL systems of interest, in situ and in operando.

## Supporting Information Available

Sample preparation and characterization; spectral line shape fitting and kinetic modeling; transient data for DPTS/EAF-3D and HPTS/acetate (1 M) in D_2_O; and p*K*_a_ and p*K*_a_* determination (PDF)
Transparent Peer Review report available (PDF)
