# Supplementary material for: pH Jumps in a Protic Ionic Liquid Proceed by Vehicular Proton Transport
Source: J Phys Chem Lett. 2022 Aug 23;13(34):8104–10. doi: 10.1021/acs.jpclett.2c01457 (PMC9442784; doi:10.1021/acs.jpclett.2c01457)
Supplement: Supplementary file 1 — jz2c01457_si_001.pdf [file jz2c01457_si_001.pdf]

pH-jumps in a Protic Ionic Liquid Proceed by Vehicular Proton Transport

Sourav Maiti[§], Sunayana Mitra[¶], Clinton A. Johnson[¶,$], Kai C. Gronborg[¶], Sean Garrett-Roe[¶*] and Paul M. Donaldson[§*]

[§]*Central Laser Facility, RCaH, STFC-Rutherford Appleton Laboratory, Harwell Science and Innovation Campus, Didcot, OX11 0QX, United Kingdom*

[¶]*Department of Chemistry, University of Pittsburgh, 219 Parkman Avenue, Pittsburgh, Pennsylvania 15260, United States*

*Corresponding Authors

Sean Garrett-Roe: sgr@pitt.edu

Paul M. Donaldson: paul.donaldson@stfc.ac.uk

[$]Current address: *Department of Chemistry, Davis and Elkins College, 100 Campus Dr., Elkins, WV 26241, USA*

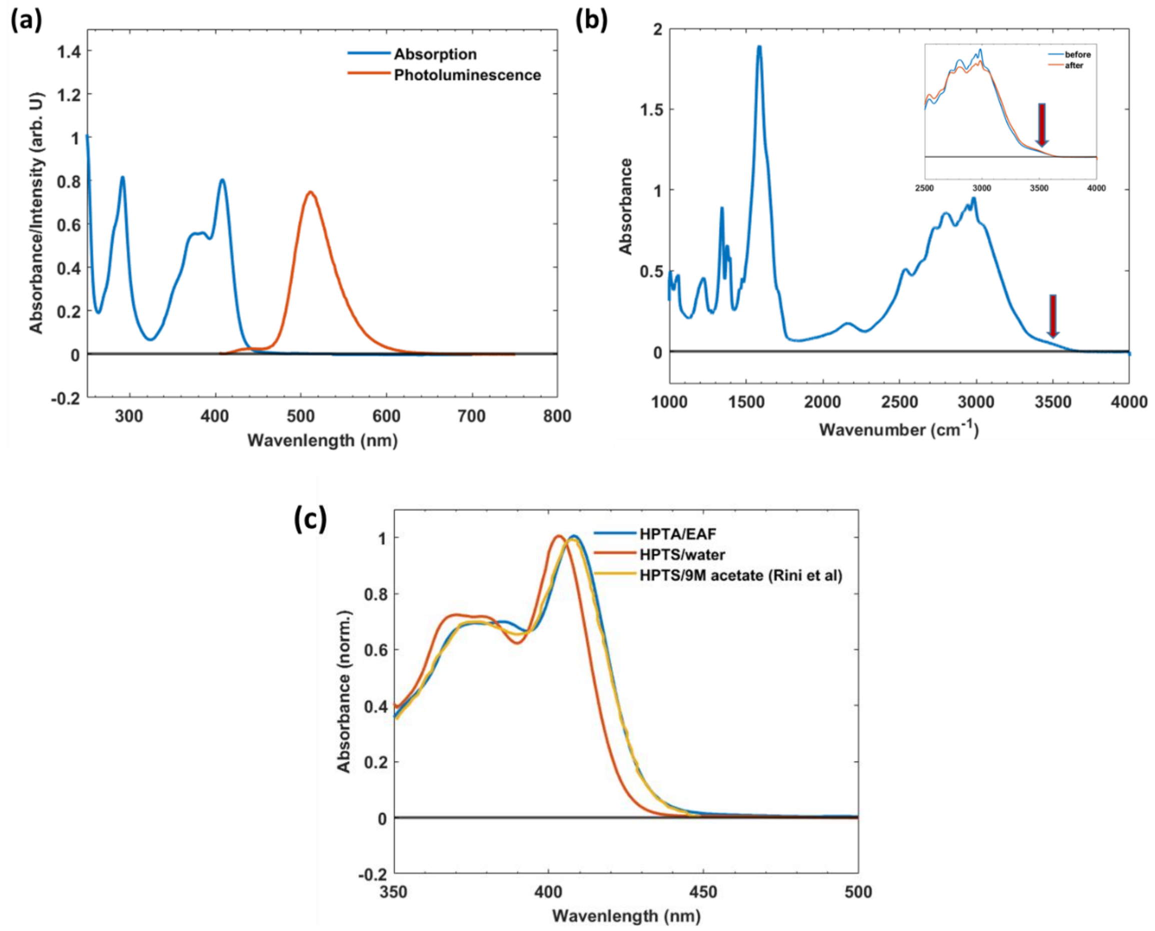

Figure S1. (a) UV-vis absorption and photoluminescence (400 nm excitation) spectra of HPTS/EAF. The photoluminescence spectrum shows RO^{*-} emission at ~510 nm is predominant. (b) FTIR spectrum of EAF/HPTS. The arrow ~3500 cm^{-1} shows small (< 1 wt %) water content. (Inset) FTIR before and after TRMPS experiment showing water content remains the same. (c) Comparison of absorption spectra for HPTS in water, EAF and 9M aqueous acetate solution (data from Rini et al. J. Chem. Phys. 2004, 121, 9593).[1] The plot shows ~4 nm redshift of HPTS absorption maxima in EAF. The absorption spectra of HPTS in EAF and 9M aqueous acetate are similar. The concentration of formate in EAF is 11.4 M (see section d). Because of the high acceptor concentration in both EAF and 9M aqueous acetate the absorption spectra are quite similar.

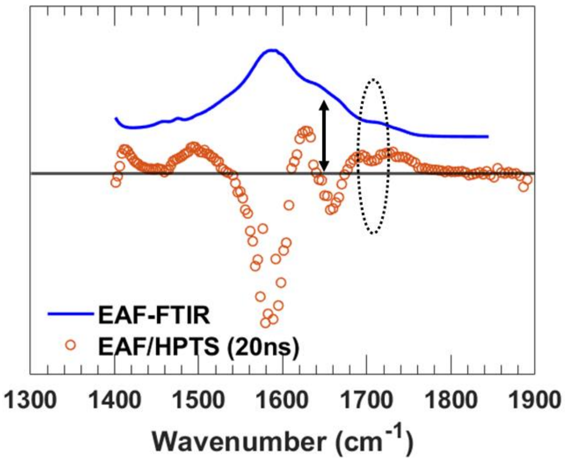

Figure S2. An EAF FTIR spectrum and a transient absorption spectrum of HPTS/EAF at 20 ns delay. The apparent split around 1710 cm^{-1} in the formic acid band is due to an EAF band around the same region.

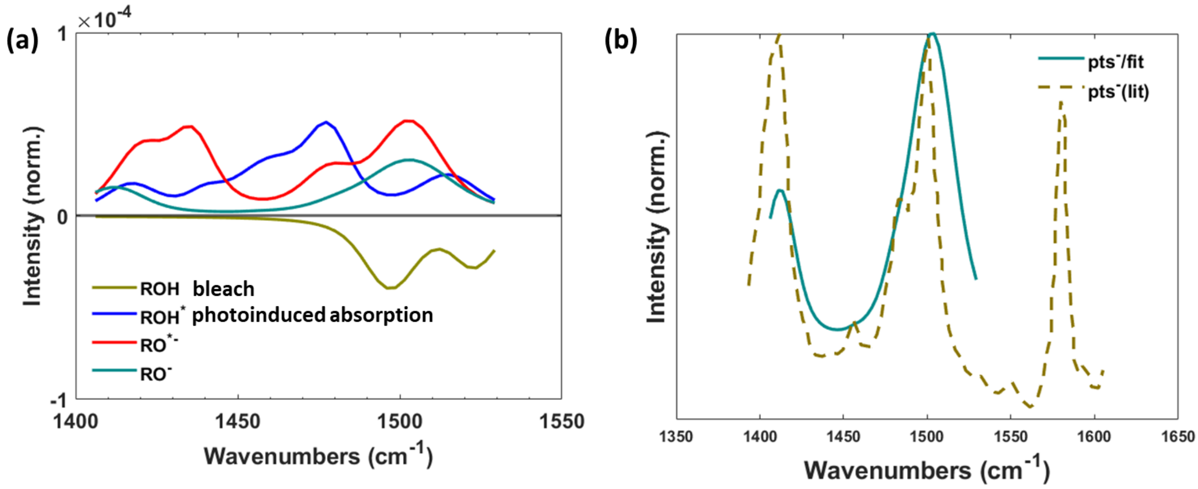

Figure S3. (a) The spectra of ROH, ROH*, RO^{*-} and RO^{-} as determined from Voigt profile analysis. (b) Comparison of Voigt spectra of PTS^{-} with literature[2].

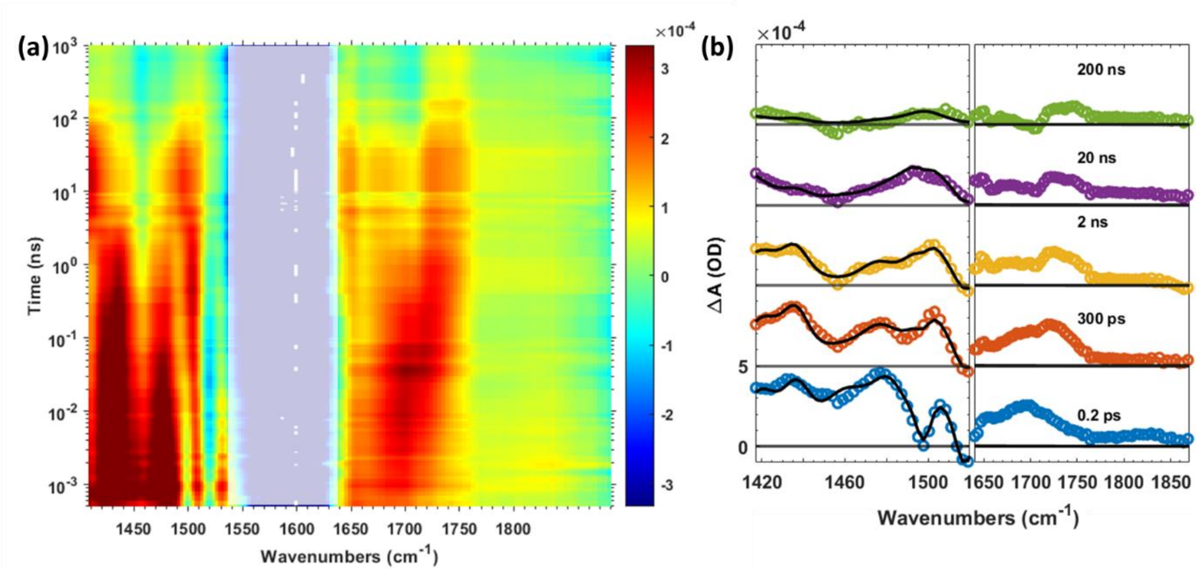

Figure S4. (a) 2D colourmap representing the transient absorbance (ΔA) of 20 mM DPTS dissolved in deuterated ethylammonium formate (EAF-3D) upon 400 nm pump excitation (fwhm ~150 fs). (b) Fitting the transient spectra (data points) with Voigt profile (solid line) of ROH, ROH^*, ROH^{*-} and ROH^- at representative delay times in DPTS/EAF-3D.

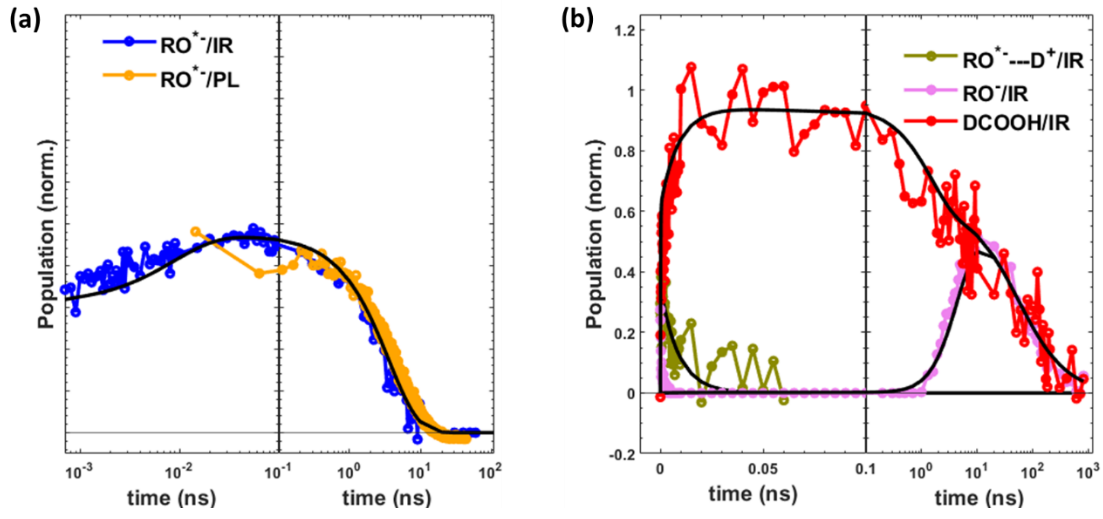

(c)

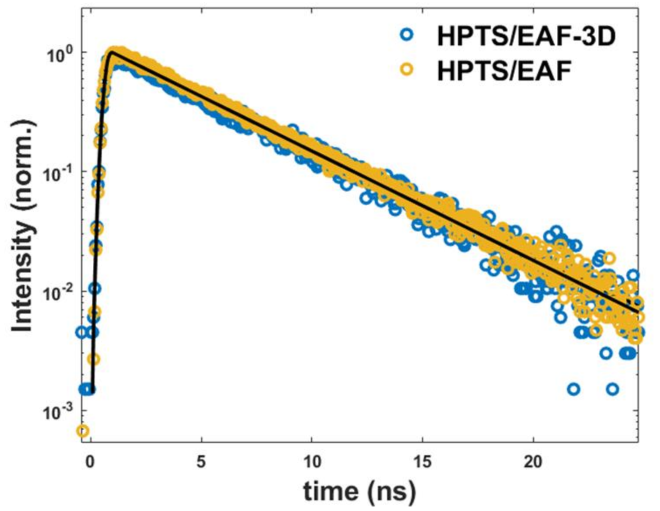

Figure S5. (a) Kinetics of RO^{*-} and the photoluminescence (PL) decay in DPTS/EAF-3D. (b) Kinetics of loosely bound protons (from an average of signal across 1770-1850 cm^{-1}), RO^{-} and formic acid (average across the region 1720-1735 cm^{-1}). The kinetics for RO^{*-} and RO^{-} are obtained from spectral model fitting of transient infrared absorption data. The black lines represent fits according to the kinetic model in Figure 1. (c) The photoluminescence decay comparison of HPTS/EAF and HPTS/EAF-3D upon 405 nm photoexcitation. The decay was fitted with a single exponential (black lines) and $KIE = {}^{k_H}/_{k_D}$ ~1 was obtained.

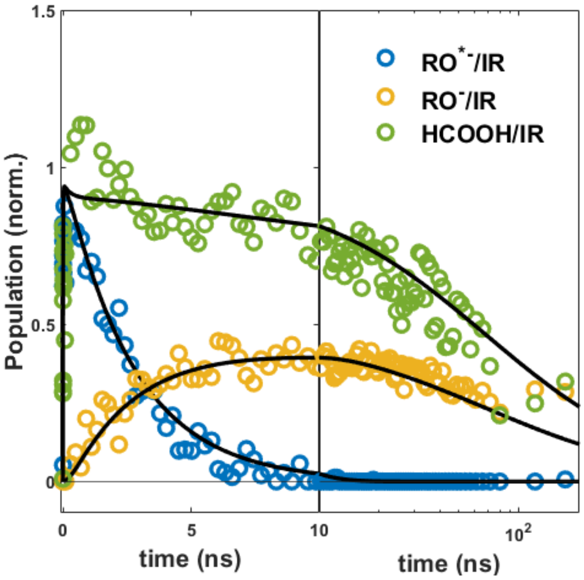

Figure S6. Extracted kinetics of RO*- and RO- from the spectral model fitting of transient data and kinetics of acetic acid for HPTS/1M sodium acetate/D_2O. Black lines are the fits according to the kinetic model (scheme 1 and section 3, SI).

Table S1. Fitted rate constants for HPTS/1M sodium acetate/D_2O.[a]

Rate constants	HPTS/acetate (1M)/D_2O
$\boldsymbol{k_{PT}}$	$(150\ fs)^{-1}$
$\boldsymbol{k_{PT_s}}$	$113.2\ \pm 6.1\ ns^{-1}$
$\boldsymbol{k_{Rec}}$	$399.9\ \pm 21.0\ ns^{-1}$
$\boldsymbol{k_{Diss}}$	$3.4 \pm 0.2\ ns^{-1}$
$\boldsymbol{k_a}$[b]	5×10^{10} M^{-1}s^{-1}
$\boldsymbol{k_{PL}}$	$0.37 \pm .01\ ns^{-1}$
$\boldsymbol{k'_a}$	$(1.57 \pm 0.10) \times 10^{10}$ M^{-1}s^{-1}
$\boldsymbol{k'_{Rec}}$	$150.1 \pm 34.8\ ps^{-1}$

[a] Rate constants are optimized within acceptable values as reported in the literature.[1-11] [b] Fixed rate constant taken from ref. [9] was used.

1. **Experimental section**

a. **Chemicals:**

HPTS (Sigma Aldrich,$\geq$ 97%), Ethylamine (70 %wt in water, Sigma Aldrich), Formic acid (Sigma Aldrich), sodium acetate (Sigma Aldrich), D_2O (99.9 atom%, Sigma Aldrich) were used as received.

b. **Preparation of samples**

EAF was synthesized by titrating ethylamine with formic acid in an equimolar ratio at -70°C. The synthesized EAF was Schlenk vacuum line dried for seven hours to reduce the water content below 1 wt%. The EAF formation was confirmed through FTIR and ^{1}H-NMR. The EAF was dried a second time prior to performing the TRMPS experiments.

Deuteration was performed by placing the dry, newly synthesized EAF with 5 times excess of D_2O in a flask. The solution was held at ~35 °C and stirred for ~12 hours to ensure full exchange. A vacuum pump setup removed the heavy water in the solution. This deuteration process was repeated three times to ensure full labile H to D exchange, giving the desired EAF-3D. FTIR and ^{1}H NMR spectroscopies characterized the new compounds. In FTIR spectroscopy, following the ND/NH stretch peak ratio throughout the exchange shows an increasing ND band (~2200 cm^{-1}) intensity to over 98% of the NH band (~3000 cm^{-1}). NMR spectroscopy was used to monitor the disappearance of the NH_3 peak to ensure complete H/D exchange. At the end of the exchange, the PILs were again dried at 65-70 °C using a vacuum pump setup, and the decreasing OD stretch of D_2O (~2500 cm^{-1}) intensity in an FTIR spectrometer was checked.

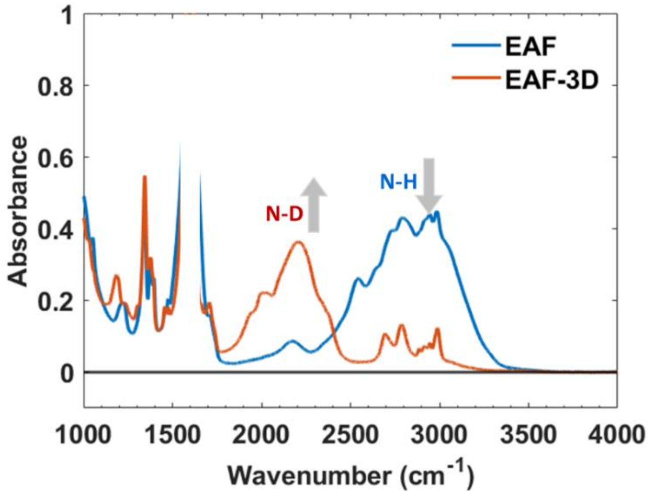

Figure S7. FTIR spectra of EAF and EAF-3D.

The FTIR spectrum in Figure S7 shows the appearance of an N-D stretching band at the expense of the N-H stretching band, confirming the H/D exchange. The around region ~1570 cm^{-1} is excluded from the plot due to saturation of the acid carbonyl band.

20 mM solutions of HPTS in EAF were prepared in a glovebag filled with dry N_2. For aqueous experiments, 20mM of HPTS was dissolved in D_2O with 1M sodium acetate. For FTIR and TRMPS measurements, the samples were placed in a Harrick cell with CaF_2 windows and no spacer. The Harrick cell was assembled inside a dry N_2-filled glovebag.

The acid-base reaction in a protic ionic liquid can be represented as:

$$HA + B \rightleftarrows BH^{+} + A^{-}$$

The degree of ionization has been estimated from pK_S where $pK_S = -log_{10}K_s = -\log([HA][B])$.

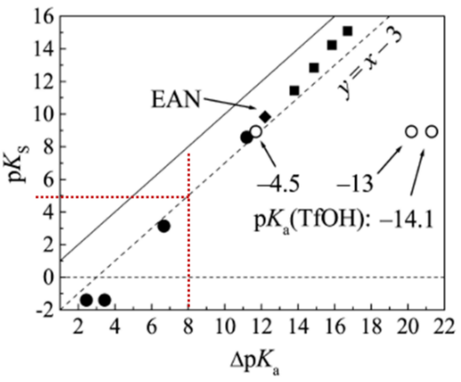

Figure 6. Relationship between ΔpK_a and pK_S in the present PILs. The closed diamonds are for EAN[27] and a series of PILs composed of a ternary ammonium with 2-hydroxyethyl group(s).[56].

From Kanzaki, R.; Doi, H.; Song, X.; Hara, S.; Ishiguro, S.-i.; Umebayashi, Y., Acid–Base Property of N-Methylimidazolium-Based Protic Ionic Liquids Depending on Anion. *J. Phys. Chem. B* **2012**, *116*, 14146-14152.

The pK_S in ethylammonium formate is ~5 ($\Delta pK_a = pK_a(HB^+) - pK_a(A^-) = 7.8$) which gives the ethylamine concentration of $3 \times 10^{-3} M$. The concentration of ionic species $c_i = 1000 * \frac{density}{molecular\ weight} = 11.4\ M$. Therefore, in an equimolar mixture of ethylamine and formic acid, the ratio of ethylammonium to neutral amine is ~3800:1. This suggests proton transfer from photoexcited HPTS to ethylamine is practically negligible.

c. Photoluminescence lifetime measurements:

Photoluminescence lifetimes were obtained using the time-correlated single-photon counting (TCSPC) method on a home-built instrument based at RAL’s CLF-Octopus facility. The HPTS was excited with 800 nm laser pulses (~100 ps), and two-photon absorption resulted in the emission from the photoexcited species around 510 nm.

d. Data analysis:

Data plotting and analysis were conducted in LABVIEW and MATLAB (R2021a).

2. Voigt profile analysis of transient absorption data:

To extract the time-dependent concentrations of the components of the photoprotolytic cycle of HPRS/EAF from the TR-IR data, we initially attempted singular value decomposition (SVD) to identify different components with distinguishable time-dependence. Significant overlap in the transient IR spectra between the different relevant species which co-exist together (ROH* and especially RO^{*-} and RO^{-}) for a considerable period of time delay resulted in the SVD analysis giving components that did not separate into individual chemical species. The transient spectra at each delay time were instead fitted with model spectral profiles of ROH, ROH^{*}, RO^{*-} and RO^{-}.

Voigt lineshapes were used for the spectral fitting.[12] Voigt lineshapes are a convolution of Gaussian and Lorentzian distributions. In the absence of detailed IR lineshape information for the ROH, ROH^{*}, RO^{*-} and RO^{-} species, Voigt lineshape fits encompass both forms of broadening.

The Voigt lineshapes are defined from a Lorentzian lineshape with a centre at $\hat{\nu}$ and width of γ_L :

$$g_L(\nu - \hat{\nu}, \gamma_L) = \frac{\gamma_L/\pi}{(\nu - \hat{\nu})^2 + \gamma_L^2}$$

A Gaussian lineshape with centre at $\hat{\nu}$ and width of γ_G:

$$g_G(\nu - \hat{\nu}, \gamma_G) = \frac{1}{\gamma_G}(\frac{ln2}{\pi})^{\frac{1}{2}}\exp[-ln2(\frac{\nu - \hat{\nu}}{\gamma_G})^2]$$

The Voigt lineshape is then calculated as:

$g_V(\nu - \hat{\nu}, \gamma_{G,}\gamma_L) = g_L \otimes g_G$, where $\otimes$ refers to convolution.

At early times (<1 ns), the dominating species in transient spectra are ROH* and RO^{*-}, whereas at very long delay times (>10 ns) the only species contributing is RO^- therefore the transient spectra at extreme times were used as a guide to determine the Voigt profiles of these species.

3. Kinetic scheme analysis:

Scheme S1 describes the full photoprotolytic cycle of HPTS.

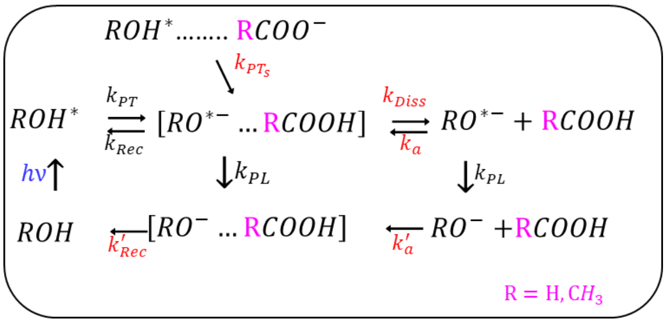

Scheme S1. A proposed photoprotolytic cycle of HPTS in water/acetate and EAF.

The rates of all the steps involved can be written as follows:

$$\frac{d[ROH^*]_1}{dt} = -k_{PT}\varphi_1[ROH^*] + k_{Rec}[RO^{*-} \ldots RCOOH] \tag{1}$$

$$\frac{d[ROH^*]_2}{dt} = -k_{PT_s}\varphi_2[ROH^*] \tag{2}$$

$$\frac{d[RO^{*-} \ldots RCOOH]}{dt} = (k_{PT}\varphi_1 + k_{PT_s}\varphi_2)[ROH^*] - (k_{diss} + k_{Rec} + k_{PL})[RO^{*-} \ldots RCOOH] + k_a[RO^{*-}][RCOOH] \tag{3}$$

$$\frac{d[RO^{*-}]}{dt} = k_{Diss}[RO^{*-} \ldots RCOOH] - k_a[RO^{*-}][RCOOH] - k_{PL}[RO^{*-}] \tag{4}$$

$$\frac{d[RO^-]}{dt} = k_{PL}[RO^{*-}] - k'_a[RO^-][RCOOH] \tag{5}$$

$$\frac{d[RCOOH]}{dt} = k_{Diss}[RO^{*-} \ldots RCOOH] - k_a[RO^{*-}][RCOOH] - k'_a[RO^-][RCOOH] \tag{6}$$

$$\frac{d[RO^- \ldots RCOOH]}{dt} = k'_a[RO^-][RCOOH] + k_{PL}[RO^{*-} \ldots RCOOH] - k'_{Rec}[RO^- \ldots RCOOH] \tag{7}$$

$$\frac{d[ROH]}{dt} = k'_{Rec}[RO^- \ldots RCOOH] \tag{8}$$

φ_1 *and* φ_2 *refers to proportion of tight and loose complex, respectively.*

The coupled differential equations (1) – (8) were solved in Matlab (version R2021a) using solver "ode23s". The loosely bound proton population (RO---H^{*-}), RO^{*-}, RO^- and formic acid

populations were simultaneously fit through least-square analysis ("lsqcurvefit" in Matlab). We used "Multistart" (100 runs) to search for a global minimum during fitting. The standard errors were obtained through the bootstrapping method (resampling residuals for 100 runs). The overall χ^2 values for HPTS/EAF system and DPTS/EAF (3D) system were 3.25 and 3.01, respectively.

4. Diffusion length analysis:

The diffusion length as described in the main text is estimated as follows:

The diffusion constant of Formic acid in water: D_{HCOOH}=1.5x10^{-9} m^2/s

Diffusion coefficient of HPTS [13] (ROH) in water D_{ROH}=3.3x10^{-10} m^2/s

The diffusion of the two species increases the total amount of diffusion:

$$D = D_{HCOOH} + D_{RO^-}$$

We assume $D_{RO^-} \sim D_{ROH}$

Given the net diffusion of the two species *D*, the average separation of the two species developing on time τ is:

$$L_D = \sqrt{D\tau}$$

The viscosity (η) for water: 1milliP.s   EAF: 23.1 milliP.s[14]

Using Stokes-Einstein relation $D \propto \frac{1}{\eta}$

Therefore, D in EAF=8x10^{-11} m^2/s

L_D in EAF: L_D= 4.9 nm.

1 solvation shell is approx. 0.5 nm. [15]

Therefore the diffusion is about 8-10 solvation shells.

5. Estimation of ground and excited state pK_a of HPTS in EAF

We have approximately estimated the ground and excited-state pK_a of HPTS in EAF from the steady-state absorption and photoluminescence spectra. The pK_a of HPTS in water is reported at ~7.4.[6] The excited-state pK_a (pK_a^*) is reported as ~0.4 in some publications

whereas others report values ~1 and ~1.4.[16-22] However, the change in pK_a ($\Delta pK_a = pK_a - pK_a^*$) upon photoexcitation is ~6 units indicating the proton-transfer in photoexcited HPTS is highly favourable to a suitable acceptor. We estimate the pK_a and pK_a^* of HPTS in water ~7.8 (Figure S8, SI) and 1.5 (Figure S9, SI), respectively indicating ΔpK_a~6.3. For HPTS in EAF the pK_a and pK_a^* values are ~8.9 and 2.5 indicating ΔpK_a~6.4.

Umebayashi and co-workers have reported pK_a of several molecules in an analogous protic ionic liquid ethylammonium nitrate (*Angew. Chem.* 2016, 128, 6374-6377) through potentiometric titrations with the electromotive force (emf) measurements.[23] They concluded pK_a in EAN (pK_a^{EAN}) is about 1 unit greater from pK_a in water ($pK_a^{EAN} = pK_a^{Water} + 1$). Our results also indicate that the pK_a values are within 1 unit in EAF compared to water.

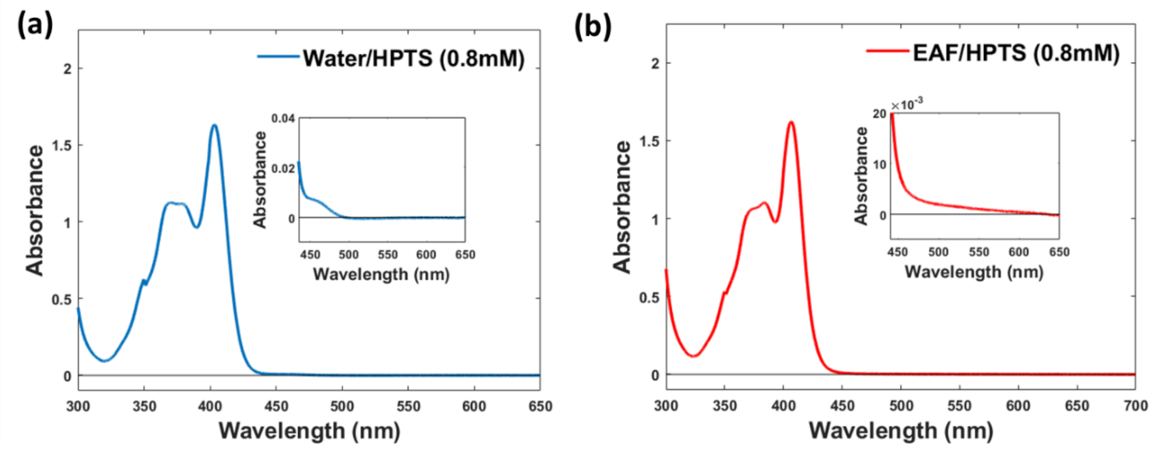

Figure S8. *(a) Absorption spectra of HPTS in water for 0.8 mM solution. (b) Absorption spectra of HPTS in EAF for 0.8 mM solution. HPTS ground state peak absorption is ~ 404 nm. The shoulder absorption of ~ 460 nm is due to RO⁻. From the absorbance ratio of ROH and RO⁻ the* pK_a *was approximately calculated. In EAF, the HPTS absorption maxima shifts to ~408 nm.*

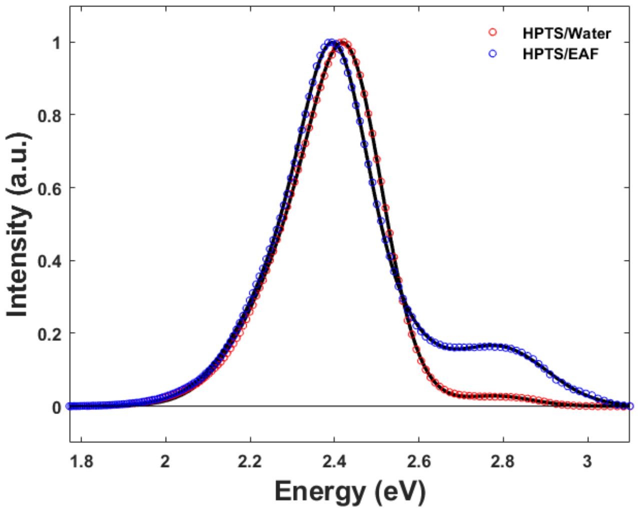

Figure S9. Photoluminescence spectra of HPTS in EAF and water (~0.8 mM). The spectra were fitted with the sum of Gaussian functions (shown as black solid lines) to obtain the area for RO^{*-} emission (~520 nm, 2.4 eV) and RO^{*} emission (~440 nm, 2.8 eV). From the area ratio of RO^{*-} and RO^{-} the pK_a^* was approximately calculated to be ~ 1.5 and ~ 2.5 for HPTS in water and EAF, respectively.

The ΔpK_a can also be estimated from absorption and emission maxima through the following equation:[18]

$$\Delta pK_a = pK_a - pK_a^* = (E_{HPTS} - E_{PTS^-})/2.3RT$$

R is the ideal gas constant (8.3 J/K·mol) and T is the temperature in K.

where the energies of individual species can be estimated as

$$E_i = Nhc(\frac{\overline{\nu_A} + \overline{\nu_F}}{2})$$

$\overline{\nu_A}$ and $\overline{\nu_F}$ represents the absorption and emission maxima in cm^{-1}, respectively.

N is Avogadro's number, h is the Planck constant, c is the speed of light.

Figure S9 shows the absorption and emission spectra of HPTS in water for 0.8 mM and 8 mM solution. From the dilute solution, the absorption and emission maxima of undissociated acid

(HPTS) can be determined. From the concentrated solution absorption and emission maxima of photobase (PTS^-) can be determined.

ΔpK_a for HPTS in water was estimated to be ~6.3 in agreement with the literature.

In the same way, ΔpK_a for HPTS in EAF was estimated to be ~6.7 agreeing with our previous measurement (Figure S9).

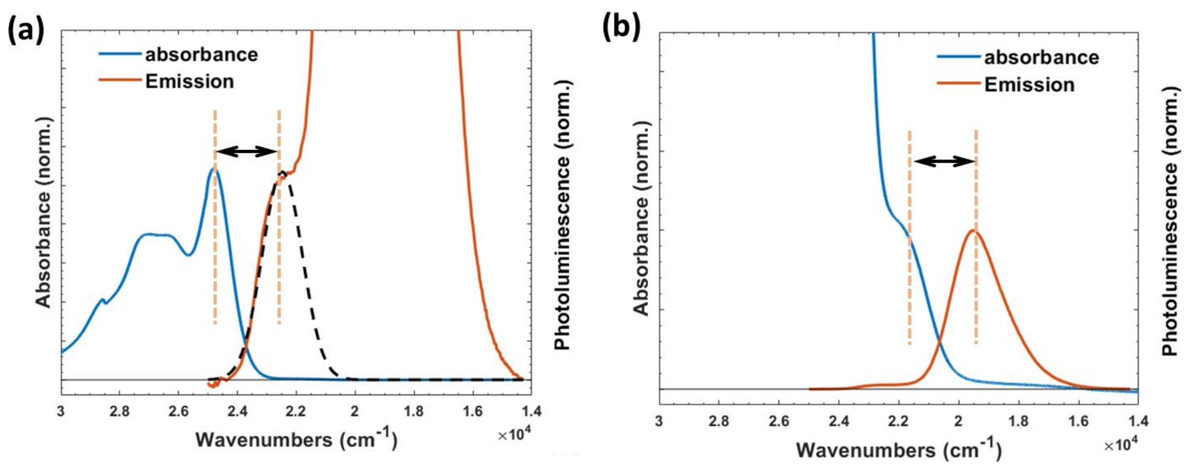

Figure S9. Absorbance and photoluminescence spectra of HPTS in water showing the absorption and emission maxima for (a) ROH (HPTS) and (b) RO^- (PTS^-). The dotted black line in (a) represents the Gaussian to fit the emission peak.

Discussion about pK_a of formic acid in EAF:

Formate is the proton acceptor from photoexcited HPTS (ROH^*) in EAF. The pK_a of formic acid in water is 3.7. We estimated pK_a^* of HPTS in EAF ~2.3 which makes the proton transfer to formate feasible. However, the pK_a of formic acid will certainly change in EAF and determination of pK_a in a protic ionic liquid media is an active area of research. As mentioned above, in the case of ethylammonium nitrate (EAN) the pK_a was 1 unit higher than water for several molecules ($pK_a^{EAN} = pK_a^{Water} + 1$).[23] Lemordant and co-workers reported that in diisopropylethylammonium formate (DIPEF) the pK_a of formic acid was found to be ~5.4,

almost two units higher than pK_a in water.[24] As ethylammonium formate (EAF) is structurally similar to DIPEF, we expect that the pK_a of formic acid in EAF will be higher (within two pK_a units) than pK_a in water. This suggests the proton transfer from photoexcited HPTS (ROH^*) to formate will be favourable in EAF.

References:

1. Rini, M.; Pines, D.; Magnes, B. Z.; Pines, E.; Nibbering, E. T., Bimodal Proton Transfer in Acid-Base Reactions in Water. *J. Chem. Phys.* **2004**, *121*, 9593-9610.
2. Mohammed, O. F.; Pines, D.; Dreyer, J.; Pines, E.; Nibbering, E. T., Sequential Proton Transfer through Water Bridges in Acid-Base Reactions. *Science* **2005**, *310*, 83-6.
3. Sudip Kumar Mondal, K. S., Subhadip Ghosh, Pratik Sen, and Kankan Bhattacharyya, Excited-State Proton Transfer from Pyranine to Acetate in Γ-Cyclodextrin and Hydroxypropyl Γ-Cyclodextrin. *J. Phys. Chem. A* **2006**, *110*, 13646.
4. Mohammed, O. F.; Dreyer, J.; Magnes, B. Z.; Pines, E.; Nibbering, E. T., Solvent-Dependent Photoacidity State of Pyranine Monitored by Transient Mid-Infrared Spectroscopy. *Chemphyschem* **2005**, *6*, 625-36.
5. Mohammed, O. F.; Pines, D.; Nibbering, E. T.; Pines, E., Base-Induced Solvent Switches in Acid-Base Reactions. *Angew. Chem. Int. Ed. Engl.* **2007**, *46*, 1458-61.
6. Mohammed, O. F.; Pines, D.; Pines, E.; Nibbering, E. T. J., Aqueous Bimolecular Proton Transfer in Acid–Base Neutralization. *Chem. Phys.* **2007**, *341*, 240-257.
7. Rini, M.; Magnes, B.-Z.; Pines, E.; Nibbering, E. T. J., Real-Time Observation of Bimodal Proton Transfer in Acid-Base Pairs in Water. *Science* **2003**, *301*, 349-352.
8. Genosar, L.; Cohen, B.; Huppert, D., Ultrafast Direct Photoacid−Base Reaction. *J. Phys. Chem. A* **2000**, *104*, 6689-6698.
9. Pavel Leiderman, L. G., and Dan Huppert, Excited-State Proton Transfer: Indication of Three Steps in the Dissociation and Recombination Process. *J. Phys. Chem. A* **2005**, *109*, 5965-5977.
10. Siwick, B. J.; Cox, M. J.; Bakker, H. J., Long-Range Proton Transfer in Aqueous Acid−Base Reactions. *J. Phys. Chem. B* **2008**, *112*, 378-389.
11. Siwick, B. J.; Bakker, H. J., On the Role of Water in Intermolecular Proton-Transfer Reactions. *J. Am. Chem. Soc.* **2007**, *129*, 13412-13420.
12. Schreier, F., Optimized Implementations of Rational Approximations for the Voigt and Complex Error Function. *J. Quant. Spectrosc. Radiat. Transfer* **2011**, *112*, 1010-1025.
13. Xia, P.; Bungay, P. M.; Gibson, C. C.; Kovbasnjuk, O. N.; Spring, K. R., Diffusion Coefficients in the Lateral Intercellular Spaces of Madin-Darby Canine Kidney Cell Epithelium Determined with Caged Compounds. *Biophys. J.* **1998**, *74*, 3302-3312.
14. Shakeel, A.; Mahmood, H.; Farooq, U.; Ullah, Z.; Yasin, S.; Iqbal, T.; Chassagne, C.; Moniruzzaman, M., Rheology of Pure Ionic Liquids and Their Complex Fluids: A Review. *ACS Sustain. Chem. Eng.* **2019**, *7*, 13586-13626.
15. Hayes, R.; Imberti, S.; Warr, G. G.; Atkin, R., Effect of Cation Alkyl Chain Length and Anion Type on Protic Ionic Liquid Nanostructure. *J. Phys. Chem. C* **2014**, *118*, 13998-14008.
16. Changenet, P.; Gustavsson, T.; Lampre, I., Introduction to Femtochemistry: Excited-State Proton Transfer from Pyranine to Water Studied by Femtosecond Transient Absorption. *J. Chem. Educ.* **2020**, *97*, 4482-4489.
17. Maurer, P.; Thomas, V.; Iftimie, R., A Computational Study of Ultrafast Acid Dissociation and Acid–Base Neutralization Reactions. Ii. The Relationship between the Coordination State of Solvent Molecules and Concerted Versus Sequential Acid Dissociation. *J. Chem. Phys.* **2011**, *134*, 094505.
18. Lakowicz, J. R., Principles of Fluorescence Spectroscopy; Chapter 7. 3rd ed.; Springer-Verlag: US 2006; pp 256-276.

19. Das, I.; Panja, S.; Halder, M., Modulation and Salt-Induced Reverse Modulation of the Excited-State Proton-Transfer Process of Lysozymized Pyranine: The Contrasting Scenario of the Ground-State Acid–Base Equilibrium of the Photoacid. *J. Phys. Chem. B* **2016**, *120*, 7076-7087.
20. Solntsev, K. M.; Sullivan, E. N.; Tolbert, L. M.; Ashkenazi, S.; Leiderman, P.; Huppert, D., Excited-State Proton Transfer Reactions of 10-Hydroxycamptothecin1. *J. Am. Chem. Soc.* **2004**, *126*, 12701-12708.
21. Agmon, N.; Pines, E.; Huppert, D., Geminate Recombination in Proton-Transfer Reactions. Ii. Comparison of Diffusional and Kinetic Schemes. *J. Chem. Phys.* **1988**, *88*, 5631-5638.
22. Pines, E.; Huppert, D.; Agmon, N., Geminate Recombination in Excited-State Proton-Transfer Reactions: Numerical Solution of the Debye–Smoluchowski Equation with Backreaction and Comparison with Experimental Results. *J. Chem. Phys.* **1988**, *88*, 5620-5630.
23. Kanzaki, R.; Kodamatani, H.; Tomiyasu, T.; Watanabe, H.; Umebayashi, Y., A Ph Scale for the Protic Ionic Liquid Ethylammonium Nitrate. *Angew. Chem. Int. Ed.* **2016**, *55*, 6266-6269.
24. Anouti, M.; Caillon-Caravanier, M.; Le Floch, C.; Lemordant, D., Alkylammonium-Based Protic Ionic Liquids Part I: Preparation and Physicochemical Characterization. *J. Phys. Chem. B* **2008**, *112*, 9406-9411.
