# Supplementary material for: pH Jumps in a Protic Ionic Liquid Proceed by Vehicular Proton Transport
Source: J Phys Chem Lett. 2022 Aug 23;13(34):8104–10. doi: 10.1021/acs.jpclett.2c01457 (PMC9442784; doi:10.1021/acs.jpclett.2c01457)
Supplement: Supplementary file 2 — jz2c01457_si_002.pdf [file jz2c01457_si_002.pdf]

jz-2022-01457p.R1

Name: Peer Review Information for "pH-jumps in a Protic Ionic Liquid Proceed by Vehicular Proton Transport"

First Round of Reviewer Comments

Reviewer: 1

Comments to the Author
Photoacid research has been pursued now for more than four decades. Since the late 1980s ultrafast spectroscopy has been a popular tool to follow the elementary steps of proton transfer, which are triggered by electronic excitation of photoacid molecules. A particular branch is that of photoacid - base reactions, where the proton transfer occurs between the excited photoacid and an accepting base. This particular manuscript provides a new chapter, by following the ultrafast proton transfer dynamics using time-resolved fluorescence and UV/IR pump-probe methods. Interestingly, not only the femtosecond to nanosecond time scale is explored, but also much longer time scales up to a microsecond with which the proton transfer kinetics can be followed along the full Förster cycle of the photoacid HPTS, i.e. forward and backward transfer processes are monitored. In addition to that, the solvent medium is an ionic liquid, which in photoacid research is still rather unexplored, even though proton transport in ionic liquids is a topic of increasing interest.
This manuscript needs several amendments / adjustments before a positive publication recommendation can be provided. In detail:

a) what are the pKa values and pKa* values of HPTS and of formic acid in an ionic liquid like ethylammonium formate?.   These numbers will definitely change from the well-reported values in aqueous solution. In particular, how much would the pKa/pKa* values change affect possible proton transfer reaction rates, as well as reaction yields. In particular, when the difference in pKa/pKa* values is small, a net transfer yield will be rather a fraction of 1 at longer tens to hundreds of picosecond (or longer) time scales. See for comparison a discussion on this aspect in Ekimova et al. JACS 141 (2019) 14581 DOI: 10.1021/jacs.9b03471

2) Long time scales: proton trransfer from HPTS to formate will occur on ultrafast time scales, as the accepting base is actually part of the solvent. Hence obviously the number of "tight" contact photoacid base reaction pairs will be high, maybe close to unity. Is anything known the complexation constants of HPTS with formate in the ionic liquid medium, when HPTS is in the electronic ground state?

3) Even though proton transfer can only occur between HPTS and formate in a "tight" contact confguration (because the solvent is an aprotic solvent), the fact that besides time resolution limited components, also contributions on picosecond time scales are observed. This behavior is similar to reported dynamics of HPTS with carboxylate bases in the protic solvent water, where this piscosecond component is caused by solvent separated "loose" complexes, obeying free energy reactivity correlations.. In the current situation though there is no " solvent separated" reaction pair.. so what is causing this picosecond component in the forward proton transfer process? "Diffusional motion" may be an tentative explanation, but it may well be a small geometric rearrangement of the formate anion. Or do the positively charged counterions play a role in this? Coulombic electric fields are known to be large range, and the number of ethylammonium ions is high.

3) Do all ethylammonium ions remain in the positively charged ionic state, or is there a major equilibrium between ethylammonium and ethylamine? How many ethylamine moiecules would be able to accept a proton from HPTS? Is there a way to probe possible contributions in the measured signals from this alternative reaction partner (the discussion of the measurements is on the photoacid/photobase side (via TCSPC) and formate/formic acid (via UV/IR measurements.

4) The apparent absence of a kinetic isotope effect is interpreted as evidence for a "vehicular transport mechanism"... A proper explanation is not provided here, and this should occur before such a statement is not only a suggestion but also being used in the title of the manuscript. An observed KIE of about 1.1 is not that far off from the typically reported value of 1.4 for many transfer reactions in typically aqeous solutions. See e.g. the book contribution by Ehud Pines in Isotope effects in Chemistry and Biology (ed. Kohen and Limbach) CRC/Taylor Francis 2006, pages 451-474. It is important to determine properly the pKa*/pKa values in a medium like ethylammmonium formate, and how the behavior of KIE as function of pKa is in such a medium.

Reviewer: 2
Comments to the Author

Re: jz-2022-01457p

The manuscript reports time-resolved infrared spectra of a photoacid HPTS in an ionic liquid over a wide time range spanning femtoseconds to milliseconds to investigate the excited-state proton transfer (ESPT) dynamics including the back ESPT to complete the proton transfer cycle. Time-dependent concentrations of relevant species were determined carefully and thoroughly, and the overall kinetic scheme was established. The results are mutually consistent among different chemical species and with the results by other measurements such as time-resolved photoluminescence. The main conclusions are (1) the ESPT kinetics is inhomogeneous with <150 fs and picoseconds time constants, (2) the kinetic isotope effect is absent indicating "vehicular transfer," and (3) the spatial range of ESPT is up to 8~10 solvent shells. Nearly the same results have been reported for the HPTS in water, in particular, concerning the conclusions (1) and (2) previously by other groups. Several reports of the ESPT of HPTS in ionic liquids have also been reported. This work, however, demonstrates that the time-resolved infrared can be a viable tool for the study of ESPT. This work also established the ESPT dynamics over the full time and spatial dimensions to fully realize the proton transfer cycle triggered by photoexcitation. This information may be important towards applications such as the electrolytes for hydrogen fuel cells. Therefore, publication of the manuscript in the Journal of Physical Chemistry Letters is recommended. Minor comments are listed below.

1. An ESPT kinetic scheme was proposed, and the data were fitted to the kinetic scheme using Voigot functions to represent peaks in the infrared spectra. However, the good fit does not guarantee the validity of the kinetic scheme. Perhaps an unbiased (less-biased) fit such as the global analysis by decay-associated spectra (DAS) analysis may help.
2. Are the line-shapes from the Voigot function fits close to Lorentzian or Gaussian? Can the line-shapes give a clue on the inhomogeneous dynamics?
3. Table 1: k_{PT} in (150 fs)$^{-1}$, others are in different notation and confusing.
4. Reference 13: Please correct the author list.
5. Supporting information, page S8: single value decomposition → singular value decomposition

Dear EDITOR,

Please find our revised manuscript entitled "*pH-jumps in a Protic Ionic Liquid Proceed by Vehicular Proton Transport*" for consideration in The Journal of Physical Chemistry Letters. We thank the reviewers for useful and constructive comments which allowed us to improve our paper. Below we have answers to reviewers and changes made to the manuscript.

We hope our revised manuscript is now suitable for publication in The Journal of Physical Chemistry Letters.

Best regards, Sourav Maiti, Sean Garrett-Roe and Paul Donaldson

Reviewer: 1

Comments:

Photoacid research has been pursued now for more than four decades. Since the late 1980s ultrafast spectroscopy has been a popular tool to follow the elementary steps of proton transfer, which are triggered by electronic excitation of photoacid molecules. A particular branch is that of photoacid - base reactions, where the proton transfer occurs between the excited photoacid and an accepting base. This particular manuscript provides a new chapter, by following the ultrafast proton transfer dynamics using time-resolved fluorescence and UV/IR pump-probe methods. Interestingly, not only the femtosecond to nanosecond time scale is explored, but also much longer time scales up to a microsecond with which the proton transfer kinetics can be followed along the full Förster cycle of the photoacid HPTS, i.e. forward and backward transfer processes are monitored. In addition to that, the solvent medium is an ionic liquid, which in photoacid research is still rather unexplored, even though proton transport in ionic liquids is a topic of increasing interest.

This manuscript needs several amendments/adjustments before a positive publication recommendation can be provided. In detail:

a) What are the pKa values and pKa* values of HPTS and of formic acid in an ionic liquid like ethylammonium formate? These numbers will definitely change from the well-reported values in aqueous solution. In particular, how much would the pKa/pKa* values change affect possible proton transfer reaction rates, as well as reaction yields? In particular, when the difference in pKa/pKa* values is small, a net transfer yield will be rather a fraction of 1 at longer tens to hundreds of picosecond (or longer) time scales. See for comparison a discussion on this aspect in Ekimova et al. JACS 141 (2019) 14581 DOI: 10.1021/jacs.9b03471

Answer to the reviewer:

This is indeed an important question, as discussed in the reference mentioned. We have estimated the pK_a and pk_a^* of HPTS in water and ethylammonium formate from the steady-state absorption and photoluminescence spectra. Our estimated pK_a (~ 7.8) and pk_a^* (~ 1.5) of HPTS in water is close to the reported value in literature within error. Our estimate of pK_a and pk_a^* of HPTS in ethylammonium formate are ~8.9 and ~2.5. This suggests a ΔpK_a of around 6.5 units in EAF upon photoexcitation of HPTS. The pK_a of formic acid in EAF will certainly be higher than in water - by around two pK_a units (discussion below). This suggests proton transfer from photoexcited HPTS to formate is a favourable process in EAF. The ultrafast growth (pulse-width limited) of formic acid in our transient-infrared measurements also suggests proton transfer on ~150 femtosecond time scales, not tens of picoseconds for smaller (2-4 units) difference in pK_a and pK_a^*, as queried by the reviewer. We have added the following sentence on page 13: *'Determination of pK_a in the ground and excited state of HPTS in EAF can give an idea about proton transfer rates compared to water. From steady-state absorption and photoluminescence data, we have approximately estimated a change of 6.5 pK_a units (section 5, SI) in EAF upon photoexcitation of HPTS similar to aqueous systems. This suggests proton transfer to formate in EAF is a highly favourable process as in the case of acetate in water.'*

We have added the following discussion in the SI (section 5).

1. ***Estimation of ground and excited state pK_a of HPTS in EAF***

We have approximately estimated the ground and excited-state pK_a of HPTS in EAF from the steady-state absorption and photoluminescence spectra. The pK_a of HPTS in water is reported to be ~7.4.[6] The excited-state pK_a (pK_a^*) is reported as ~0.4 in some publications whereas others report values ~1 and ~1.4.[16-22] However, the change in pK_a ($\Delta pK_a = pK_a - pK_a^*$) upon photoexcitation is ~6-7 units indicating the proton-transfer in photoexcited HPTS is highly favourable to a suitable acceptor. We estimate (Figure S8, SI) the pK_a and pK_a^* of HPTS in water ~7.8 and 1.5, respectively, indicating $\Delta pK_a \sim 6.3$. For HPTS in EAF we have estimated pK_a and pK_a^* ~8.9 and ~2.5, respectively indicating $\Delta pK_a \sim 6.4$.

Umebayashi and co-workers have reported pK_a of several molecules in an analogous protic ionic liquid ethylammonium nitrate (Angew. Chem. 2016, 128, 6374-6377) through potentiometric titrations with the electromotive force (emf) measurements.[23] They concluded pK_a in EAN (pK_a^{EAN}) is about 1 unit greater from pK_a in water $(pK_a^{EAN} = pK_a^{Water} + 1)$. Our results also indicate that the pK_a values of HPTS are within 1 unit in EAF compared to water.

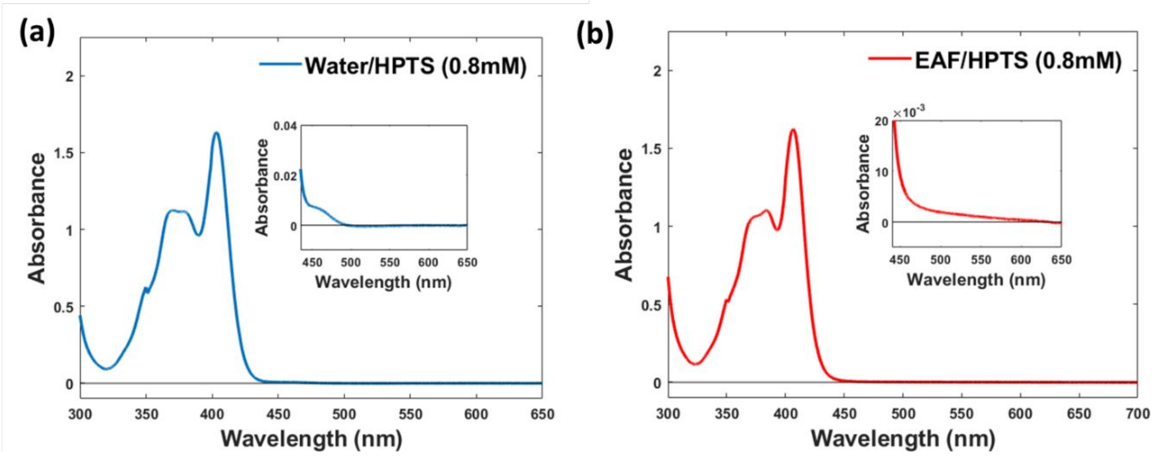

Figure S8. *(a) Absorption spectra of HPTS in water for 0.8 mM solution. (b) Absorption spectra of HPTS in EAF for 0.8 mM solution. HPTS ground state peak absorption is ~ 404 nm. The shoulder absorption of ~ 460 nm is due to RO^-. From the absorbance ratio of ROH and RO^- the pK_a was approximately calculated. In EAF, the HPTS absorption maxima shifts to ~408 nm.*

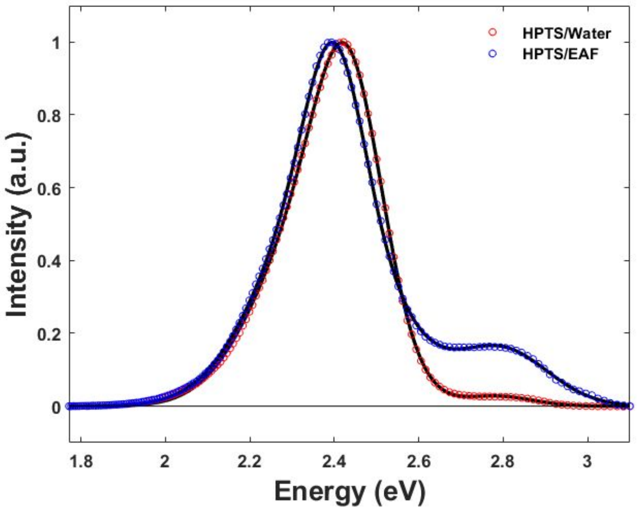

Figure S9. *Photoluminescence spectra of HPTS in EAF and water (~0.8 mM). The spectra were fitted with the sum of Gaussian functions (shown as black solid lines) to obtain the area for RO^{*-} emission (~520 nm, 2.4 eV) and RO* emission (~440 nm, 2.8 eV). From the area ratio of RO^{*-} and RO^{-} the pK_a^* was approximately calculated to be ~ 1.5 and ~ 2.5 for HPTS in water and EAF, respectively.*

The ΔpK_a can also be estimated from absorption and emission maxima through the following equation:[18]

$$\Delta pK_a = pK_a - pK_a^* = (E_{HPTS} - E_{PTS^-})/2.3RT$$

R is the ideal gas constant (8.3 J/K·mol) and T is the temperature in K.

where the energies of individual species can be estimated as

$$E_i = Nhc(\frac{\overline{\nu_A} + \overline{\nu_F}}{2})$$

$\overline{\nu_A}$ and $\overline{\nu_F}$ represents the absorption and emission maxima in cm^{-1}, respectively.

N is Avogadro's number, h is the Planck constant, c is the speed of light.

Figure S9 shows the absorption and emission spectra of HPTS in water for 0.8 mM solution.

ΔpK_a for HPTS in water was estimated to be ~6.3 in agreement with the literature and our estimation (see above).

In the same way, ΔpK_a for HPTS in EAF was estimated to be ~6.7 roughly agreeing with our previous estimation of ~6.4 (Figure S9).

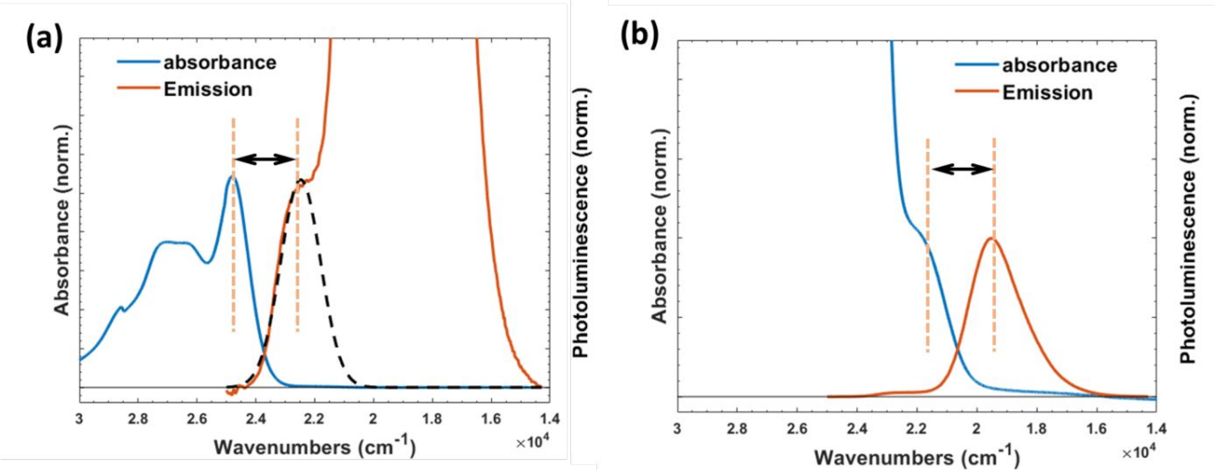

Figure S9. *Absorbance and photoluminescence spectra of HPTS in water showing the absorption and emission maxima for (a) ROH (HPTS) and (b) RO^- (PTS^-). The dotted black line in (a) represents the Gaussian to fit the emission peak.*

Discussion about pK_a of formic acid in EAF:

Formate is the proton acceptor from photoexcited HPTS (ROH^*) in EAF. The pK_a of formic acid in water is 3.7. We estimated pK_a^* of HPTS in EAF ~2.5 which makes the proton transfer to formate feasible. However, the pK_a of formic acid will certainly change in EAF and determination of pK_a in a protic ionic liquid media is an active area of research. As mentioned above, in the case of ethylammonium nitrate (EAN) the pK_a was 1 unit higher than water for several molecules ($pK_a^{EAN} = pK_a^{Water} + 1$).[23] Lemordant and co-workers reported that in diisopropylethylammonium formate (DIPEF) the pK_a of formic acid was found to be ~5.4, almost two units higher than pK_a in water.[24] As ethylammonium formate (EAF) is structurally

similar to DIPEF, we expect that the pK_a of formic acid in EAF will be higher (within two pK_a units) than pK_a in water. This suggests the proton transfer from photoexcited HPTS (ROH^*) to formate will be favourable in EAF.

2) Long time scales: proton transfer from HPTS to formate will occur on ultrafast time scales, as the accepting base is actually part of the solvent. Hence obviously the number of "tight" contact photoacid base reaction pairs will be high, maybe close to unity.

Is anything known the complexation constants of HPTS with formate in the ionic liquid medium, when HPTS is in the electronic ground state?

Answer to the reviewer:

We agree that the fraction of the tight complex will be high in EAF. From our transient infrared measurements, we have estimated that the fraction of tight complex is ~70% between ROH* and formate leading to a complexation constant of 0.21 M^{-1}. This is close to the 0.28 M^{-1} (Rini et al. *J. Chem. Phys.* 2004, 121, 9593) complexation constant of HPTS in water. We compared the steady-state absorption spectra of HPTS/water, HPTS/EAF and HPTS/9M acetate (data from Rini et al. *J. Chem. Phys.* 2004, 121, 9593) in Figure S1(c). The absorption spectrum of HPTS is 4 nm red-shifted in EAF compared to water and matches closely with the absorption spectrum of HPTS in 9M acetate. The concentration of formate in EAF is 11.4 M (*see section d, SI*). Because of the high acceptor concentration in both EAF and 9M aqueous acetate the absorption spectra are quite similar. This also indicates the 1:1 complexation of HPTS with EAF as in the case of HPTS in acetate/water.

We have added the following in the main text (page 8):

‘ *Similar to HPTS/acetate studies in water, we attribute the fast-growth component to instantaneous (pulse-width limited) proton transfer from photo-excited HPTS (ROH^*) to formate in hydrogen-bonded tight complexes pre-existing prior to photoexcitation (also supported by steady-state absorption spectra, figure S1(c), SI).[7, 22] The relative amplitude of the fast-growth component accounts for the fraction of tight-complex (~70%) from which the complexation constant[7, 22] of HPTS in EAF can be obtained as 0.21 M^{-1}.*

’

(c)

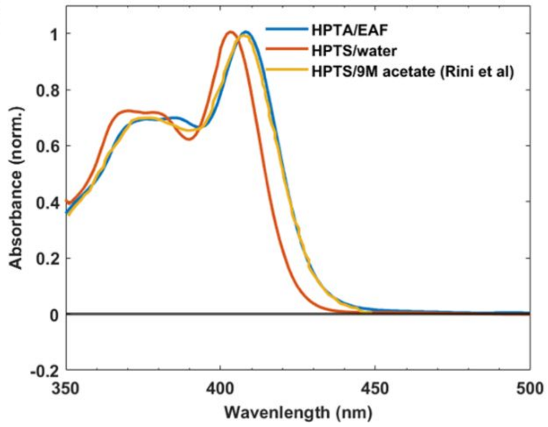

(c) Comparison of absorption spectra for HPTS in water, EAF and 9M aqueous acetate solution (data from Rini et al. J. Chem. Phys. 2004, 121, 9593).[1] The plot shows ~4 nm redshift of HPTS absorption maxima in EAF. The absorption spectra of HPTS in EAF and 9M aqueous acetate are similar. The concentration of formate in EAF is 11.4 M (see section d, SI). Because of the high acceptor concentration in both EAF and 9M aqueous acetate the absorption spectra are quite similar.

3) Even though proton transfer can only occur between HPTS and formate in a "tight" contact configuration (because the solvent is an aprotic solvent), the fact that besides time resolution limited components, also contributions on picosecond time scales are observed. This behaviour is similar to reported dynamics of HPTS with carboxylate bases in the protic solvent water, where this picosecond component is caused by solvent separated "loose" complexes, obeying free energy reactivity correlations.. In the current situation though there is no "solvent separated" reaction pair.. so what is causing this picosecond component in the forward proton transfer process? "Diffusional motion" may be an tentative explanation, but it may well be a small geometric rearrangement of the formate anion. Or do the positively charged counterions play a role in this? Coulombic electric fields are known to be large range, and the number of ethylammonium ions is high.

Answer to the reviewer:

This is an interesting aspect raised by the reviewer. The ‘loose-complex’ is not a solvent-separated encounter pair in EAF, rather we speculate, the formate and HPTS* are in a different orientation that makes the proton transfer slow. We have clarified this in the main text (page 9):

‘In water, the ‘loose-complex’ are solvent-separated encounter pairs.[7, 12-13, 22] In the case of EAF, we attribute the ‘loose-complex’ to the RO^{*-}–formate pairs with a different geometric configuration where fast proton-transfer is unfavourable. These must reorganize to form a favourable configuration prior to proton transfer. Recent theoretical investigation of direct and solvent-mediated proton transfer from HPTS in water shows the importance of structural configuration on the proton transfer rates.[35-36] Therefore, future theoretical studies in EAF can certainly help to decipher the exact configuration of loose-complex.'

The positive charge of ethylamine will help to stabilize the encounter pair and we hope our paper will attract further theoretical studies to provide more insight in future.

4) Do all ethylammonium ions remain in the positively charged ionic state, or is there a major equilibrium between ethylammonium and ethylamine? How many ethylamine molecules would be able to accept a proton from HPTS? Is there a way to probe possible contributions in the measured signals from this alternative reaction partner (the discussion of the measurements is on the photoacid/photobase side (via TCSPC) and formate/formic acid (via UV/IR measurements).

Answer to the reviewer:

The acid-base reaction in a protic ionic liquid can be represented as:

$$HA + B \rightleftarrows BH^{+} + A^{-}$$

The degree of ionization has been estimated from pK_S where $pK_S = -\,log_{10}K_s = -\log\,([HA][B])$.

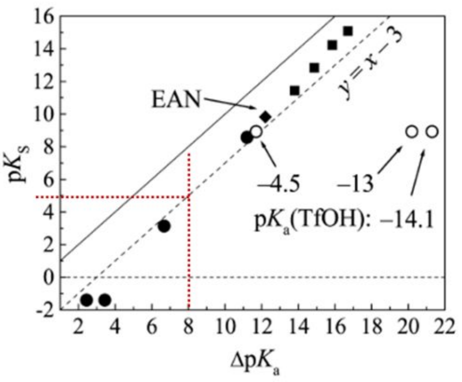

Figure 6. Relationship between ΔpK_a and pK_S in the present PILs. The closed diamonds are for EAN[27] and a series of PILs composed of a ternary ammonium with 2-hydroxyethyl group(s).[56].

From Kanzaki, R.; Doi, H.; Song, X.; Hara, S.; Ishiguro, S.-i.; Umebayashi, Y., Acid–Base Property of N-Methylimidazolium-Based Protic Ionic Liquids Depending on Anion. *J. Phys. Chem. B* **2012**, *116*, 14146-14152.

The pK_S in ethylammonium formate is ~5 ($\Delta pK_a = pK_a(HB^+) - pK_a(A^-) = 7.8$) which gives the ethylamine concentration of $3 \times 10^{-3} M$. The concentration of ionic species $c_i = 1000 * \frac{density}{molecular\ weight} = 11.4\ M$. Therefore, in an equimolar mixture of ethylamine and formic acid, the ratio of ethylammonium to neutral amine is ~3800:1. This suggests proton transfer from photoexcited HPTS to ethylamine is practically negligible.

We have added this discussion in the SI (page S8).

5) The apparent absence of a kinetic isotope effect is interpreted as evidence for a "vehicular transport mechanism"... A proper explanation is not provided here, and this should occur before such a statement is not only a suggestion but also being used in the title of the manuscript. An observed KIE of about 1.1 is not that far off from the typically reported value of 1.4 for many transfer reactions in typically aqueous solutions. See e.g. the book contribution by Ehud Pines in Isotope effects in Chemistry and Biology (ed. Kohen and Limbach) CRC/Taylor Francis 2006, pages 451-474. It is important to determine properly the pKa*/pKa values in a medium like ethylammmonium formate, and how the behaviour of KIE as function of pKa is in such a medium.

Answer to the reviewer:

In HPTS/water and HPTS/acetate systems, the published kinetic steps involving 'loose-complex' shows a KIE of 1.5 due to Grotthuss transport involving proton hopping. The

observed rate constants for EAF and EAF-3D are nearly identical within the error (Table 1) in our measurements. In the manuscript, we mentioned the KIE is less than 1.1 which we realise may cause confusion. We have corrected the paragraph in the revised manuscript (page 12):

'For HPTS in water, KIEs $k_H/k_D \sim 1.4 \sim \sqrt{m_D/m_H}$ *have been reported previously for proton transfer rate (*k_{PT_s}*) to form the 'loose-complex' in the proton transfer cycle, suggesting the Grotthuss process, for which protons transfer from donor (ROH*) to acceptor (acetate) by hopping through water wires.*[10, 12-13, 17, 20, 41-42] *The subsequent loss of the 'loose-complex' (*k_{Diss}*) also shows a KIE ~1.5. We have examined DPTS/EAF (3D), where all the exchangeable H were replaced with D, to record analogous transient absorption spectra (Figure S4-S5, SI). The kinetics of protonated and deuterated samples are nearly identical (Figure 4). The observed* k_H/k_D *is ~1 within error for all rate constants (Table 1), suggesting vehicular proton transfer and transport at all stages of the photoprotolytic cycle.'*

From our photoluminescence decay of RO^{*-} in EAF and EAF-3D, we observe a KIE of ~1 also suggesting an absence of proton-hopping during CIP* decay. Moreover, the decay of formic acid and PTS^- is identical (Figure 3(b)) suggesting a concerted process during the proton transport process. We have clarified this in the main manuscript (page 13):

'The growth of RO^{*-} *is identical to the growth of formic acid (Figure 3(a)) suggesting direct proton transfer without hopping. Moreover, the decay of formic acid and* RO^- *is identical (Figure 3(b)) indicating a concerted process during the proton transport process. We have compared the photoluminescence decay of* RO^{*-} *in EAF and EAF-3D (Figure S5(c)) showing identical decay with KIE~1 supporting the absence of proton-hopping during the CIP* decay process in EAF.'*

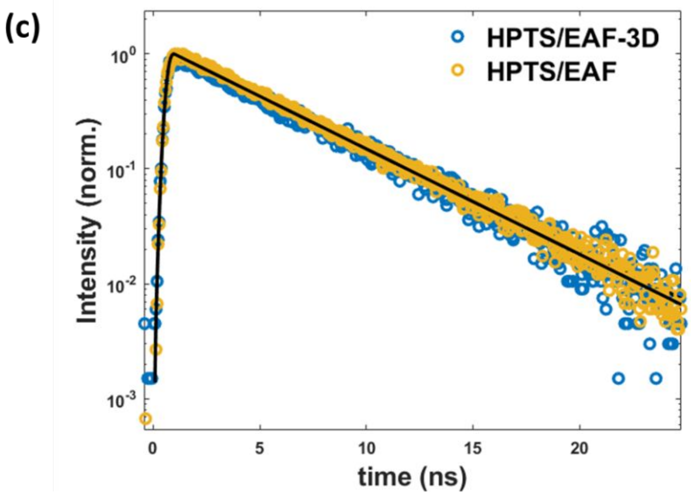

(c) The photoluminescence decay comparison of HPTS/EAF and HPTS/EAF-3D upon 405 nm photoexcitation. The decay was fitted with a single exponential (black lines) and $KIE = {}^{k_H}/_{k_D}$ ~ 1 *was obtained.'*

In aqueous systems the pk_a^* of HPTS is ~0.4-1.5. We can use a published Marcus model to infer the expected KIE to be ~3.5 during HPTS dissociation. (Pines, E., The Kinetic Isotope Effect in the Photo-Dissociation Reaction of Excited-State Acids in Aqueous Solutions. *Isotope Effects in Chemistry and Biology; Kohen A., Limbach H.-H., Eds* **2005**, 451-464.). As discussed in this reference, this was also experimentally verified for HPTS (KIE~3.2). Grotthuss proton hopping in aqueous systems have a contribution of ~1.5 ($KIE = {}^{k_H}/_{k_D} \sim 1.4 \sim \sqrt{{}^{m_D}/_{m_H}}$). We have estimated the pk_a^* of HPTS in ethylammonium formate to be ~ 2 which implies a KIE ~ 3 is expected for the dissociation of HPTS.

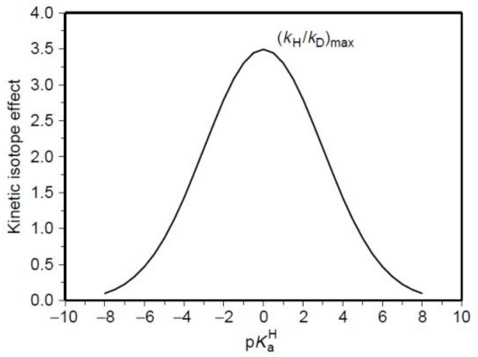

FIGURE 16.4 The functional form of the KIE predicted by the Marcus equation, Equation 16.18.

From Ehud Pines in Isotope Effects in Chemistry and Biology (ed. Kohen and Limbach) CRC/Taylor Francis 2006, pages 451-474.

Reviewer: 2

The manuscript reports time-resolved infrared spectra of a photoacid HPTS in an ionic liquid over a wide time range spanning femtoseconds to milliseconds to investigate the excited-state proton transfer (ESPT) dynamics including the back ESPT to complete the proton transfer cycle. Time-dependent concentrations of relevant species were determined carefully and thoroughly, and the overall kinetic scheme was established. The results are mutually consistent among different chemical species and with the results by other measurements such as time-resolved photoluminescence. The main conclusions are (1) the ESPT kinetics is inhomogeneous with <150 fs and picoseconds time constants, (2) the kinetic isotope effect is absent indicating "vehicular transfer," and (3) the spatial range of ESPT is up to 8~10 solvent shells. Nearly the same results have been reported for the HPTS in water, in particular, concerning the conclusions (1) and (2) previously by other groups. Several reports of the ESPT of HPTS in ionic liquids have also been reported. This work, however, demonstrates that the time-resolved infrared can be a viable tool for the study of ESPT. This work also established the ESPT dynamics over the full time and spatial dimensions to fully realize the proton transfer cycle triggered by photoexcitation. This information may be important towards applications such as the electrolytes for hydrogen fuel cells. Therefore, publication of the manuscript in the Journal of Physical Chemistry Letters is recommended.

Minor comments are listed below.

1. An ESPT kinetic scheme was proposed, and the data were fitted to the kinetic scheme using Voigt functions to represent peaks in the infrared spectra. However, the good fit does not guarantee the validity of the kinetic scheme. Perhaps an unbiased (less-biased) fit such as the global analysis by decay-associated spectra (DAS) analysis may help.

Answer to the reviewer:

Global analysis and singular Value Decomposition (SVD) are often useful for establishing kinetic schemes. We found that SVD was unable to provide feasible kinetics of the species due to overlapping spectra. To emphasize this point we have changed the sentence on page

8: *'Singular value decomposition (SVD) was applied to the kinetic scheme but failed to provide chemically meaningful components due to significant overlap in frequency and decay time of the spectra of ROH^*, RO^{*-} and RO^-, especially RO^{*-} and RO^-.'*

With SVD failing to provide meaningful results, we judged that a spectroscopic model would be necessary to obtain the kinetics of the four species. Spectral fitting with Voigt profiles provided acceptable kinetics and spectra. It is indeed important to avoid biasing the kinetic scheme with an incorrect spectroscopic model. Therefore, the Voigt spectra of individual species (ROH, ROH^*, RO^{*-}, RO^-) were constructed based on the spectra already established in the literature. We also compared the obtained kinetics with the photoluminescence decay of RO^{*-} to check the validity of the model. Moreover, we performed the Voigt spectra analysis in the water/HPTS/1M acetate system and the rate constants were quite similar to literature values, further supporting the validity of the model.

2. Are the line-shapes from the Voigt function fits close to Lorentzian or Gaussian? Can the line-shapes give a clue on the inhomogeneous dynamics?

Answer to the reviewer:

This is a really interesting question, and determining the exact specificity of the Voigt approach in comparison with Gaussian and Lorentzian fits could be a great focal point for future studies. Indeed, as the reviewer suggests, knowing about the photoacid and ionic liquid lineshapes can give clues about their solvation and dynamics. As mentioned in the text (SI Section 2), we simply chose to use Voigt lineshapes to flexibly account for lineshape variability.

3. Table 1: k_{PT} in $(150\ fs)^{-1}$, others are in different notation and confusing.

Answer to the reviewer:

To avoid confusion we have changed the unit to ns^{-1}.

4. Reference 13: Please correct the author list.

Answer to the reviewer:

We have corrected the reference.

5. Supporting information, page S8: single value decomposition → singular value decomposition.

Answer to the reviewer:

We have corrected the text.
